# What I see and what I feel: the influence of deceptive visual cues and interoceptive accuracy on affective valence and sense of effort during virtual reality cycling

**DOI:** 10.7717/peerj.16095

**Published:** 2023-10-04

**Authors:** Brendan Mouatt, Ashleigh E. Smith, Gaynor Parfitt, Ty Stanford, Jeremy McDade, Ross T. Smith, Tasha R. Stanton

**Affiliations:** 1 IIMPACT in Health, Allied Health & Human Performance, University of South Australia, Adelaide, South Australia, Australia; 2 Persistent Pain Research Group, Hopwood Centre for Neurobiology, South Australian Health and Medical Research Institute (SAHMRI), Adelaide, South Australia, Australia; 3 Alliance for Research in Exercise Nutrition and Activity (ARENA), Allied Health & Human Performance, University of South Australia, Adelaide, South Australia, Australia; 4 Clinical & Health Sciences, University of South Australia, Adelaide, South Australia, Australia; 5 Wearable Computer Laboratory, Mawson Lakes Campus, University of South Australia, Adelaide, South Australia, Australia

**Keywords:** Virtual reality, Predictive processing, Exercise, Affective valence, Ratings of perceived exertion, Perception, Interoceptive accuracy

## Abstract

**Background:**

How we feel during exercise is influenced by exteroceptive (*e.g.*, vision) and interoceptive (*i.e.*, internal body signals) sensory information, and by our prior experiences and expectations. Deceptive visual cues about one’s performance during exercise can increase work rate, without negatively impacting affective valence (good/bad responses) or perceived exertion. However, what is less understood is whether the perception of the exercise experience itself can be shifted, if work rate is held constant. Here we aimed to investigate whether deceptive vision—via illusory hills in a virtual reality (VR) cycling experience—alters affective valence and perceived exertion when physical effort is controlled. We also evaluated whether the accuracy with which one detects interoceptive cues influences the extent to which deceptive visual information can shift exercise experiences.

**Methods:**

A total of 20 participants (10 female; 30.2 ± 11.2 yrs) completed three VR cycling conditions each of 10-min duration, in a randomised, counterbalanced order. Pedal resistance/cadence were individualised (to exercise intensity around ventilatory threshold) and held constant across conditions; only visual cues varied. Two conditions provided deceptive visual cues about the terrain (illusory uphill, illusory downhill; resistance did not change); one condition provided accurate visual cues (flat terrain). Ratings of affective valence (Feeling Scale) and of perceived exertion (Borg’s RPE) were obtained at standardised timepoints in each VR condition. Interoceptive accuracy was measured *via* a heartbeat detection test.

**Results:**

Linear mixed effects models revealed that deceptive visual cues altered affective valence (*f*^2^ = 0.0198). Relative to flat terrain, illusory downhill reduced affective valence (Est = −0.21, *p* = 0.003), but illusory uphill did not significantly improve affective valence (Est = 0.107, *p* = 0.14). Deceptive visual cues altered perceived exertion, and this was moderated by the level of interoceptive accuracy (Condition-Interoception interaction, *p* = 0.00000024, *f*^2^ = 0.0307). Higher levels of interoceptive accuracy resulted in higher perceived exertion in the illusory downhill condition (*vs* flat), while lower interoceptive accuracy resulted in lower perceived exertion in both illusory hill conditions (*vs* flat) and shifts of greater magnitude.

**Conclusions:**

Deceptive visual cues influence perceptual responses during exercise when physical effort does not vary, and for perceived exertion, the weighting given to visual exteroceptive cues is determined by accuracy with which interoceptive cues are detected. Contrary to our hypotheses, deceptive visual cues did not improve affective valence. Our findings suggest that those with lower levels of interoceptive accuracy experience most benefit from deceptive visual cues, providing preliminary insight into individualised exercise prescription to promote positive (and avoid negative) exercise experiences.

## Introduction

Exercise experiences have important influences on exercise behaviour (Fox & Bailenson, 2009; Tabor et al., 2019; Williams et al., 2008b). For example, feel good or feel bad responses (affective valence) during exercise predict future exercise behaviour (Rhodes & Kates, 2015). Such findings are consistent with hedonic theory (Higgins, 2006), with individuals more inclined to do activities they enjoy, and avoid activities they do not (Williams, 2008a).

How we feel during exercise is informed by internal (interoceptive) and external (exteroceptive) sensory information, as well as our prior experiences, expectations, and beliefs (Chater et al., 2010; Friston, 2010; Knill & Pouget, 2004; Körding et al., 2007; Tabor et al., 2017). Interoceptive information may include proprioceptive/nociceptive signals from the exercising muscles, and mechanical pressure signals (specific to blood pressure and heart rate) from baroreceptors within our arteries (Garfinkel & Critchley, 2016; Tsakiris, Jiménez & Costantini, 2011). Exteroceptive information may include sensory cues about the exercising environment, such as vision of hills or the sound of cheering fans, and when combined with prior experiences, shapes our expectation of the exercise effort required. Such expectations are then checked against incoming interoceptive information, the result of which informs how we feel, allowing predictions about the consequences of our actions (Friston, 2010), and supporting behavioral regulation to maintain homeostasis (Seth & Friston, 2016).

Visual exteroceptive cues have been shown to positively influence affective responses and perception of exertion during exercise *via* attentional mechanisms, *e.g.*, distraction *via* immersive virtual reality (Jones & Ekkekakis, 2019), and *via* dissociation from exercise efforts through exergaming (Glen et al., 2017). Purposeful manipulation of visual exteroceptive cues during exercise shows that physiological information and perceptual responses are not isomorphic. That is, a change in one does not necessarily mean a similar change in the other. For example, providing deceptive cycling avatars at 102% and 105% of past performance improves current performance while preserving affect and perceived exertion (Williams et al., 2015). Thus, despite increasing incoming physiological information (relative to past performance), affect and perceived exertion are unchanged.

Whether shifts in perceptual responses can be induced by deceptive visual exteroceptive cues when the ability to regulate effort is *not* permitted is unknown but would be predicted by theories of perception based on the free energy principle (Friston, 2010) such as Bayesian predictive processing. Bayesian predictive processing posits perception, cognition, and affect as a process of probabilistic inference and prediction (Andersen et al., 2022; Seth & Friston, 2016). Prior knowledge and sensory information are combined to update beliefs and generate predictions about the environment. Thus, holding physical effort constant while providing deceptive visual cues to manipulate elements of the exercise environment that provide the user with information regarding consequences for expected effort (*e.g.*, presence/steepness of viewed hills) would be one way to test this hypothesis. From this homeostatic perspective, if an activity is more challenging than expected, and there is a discrepancy between the predicted and actual sensory information, free energy (*i.e.,* entropy or uncertainty) increases (Kiverstein, Miller & Rietveld, 2019). It is proposed that in accordance with the free energy principle, our fundamental need for survival compels us to minimise entropy, which can be achieved through affective domains like negative affect (Barrett & Simmons, 2015). Negative affect can further influence our behaviour by slowing down activity or stopping exercise.

In addition to visual information about the exercise environment, the ability to accurately detect changes in internal body signals also appears important to exercise experiences. High interoceptive accuracy is thought to enhance prediction about the body, allowing precise regulation of behavior to combat homeostatic threat (Craig, 2004; Critchley & Harrison, 2013; Herbert, Ulbrich & Schandry, 2007; Morris, 2002; Tabor et al., 2019). Interoceptive accuracy differs markedly between individuals (Craig, 2003; Garfinkel et al., 2015) and those with higher interoceptive accuracy (as assessed *via* reduced errors in heartbeat detection), demonstrate enhanced regulation of exercise effort during self-paced exercise than those with lower interoceptive accuracy (Herbert, Ulbrich & Schandry, 2007). Interoceptive accuracy may also influence the relative weighting given to incoming sensory cues, with exteroceptive cues having less influence when interoceptive accuracy is high (Seth & Friston, 2016). People with lower interoceptive accuracy experience larger exteroceptive cue-induced shifts in body perception than those with higher interoceptive accuracy (Tsakiris, Jiménez & Costantini, 2011) although this has not been evaluated within an exercise context.

We investigated the influence of deceptive exteroceptive visual cues about the exercise environment, and their interaction with interoceptive accuracy, on affective valence and perceived exertion during exercise. While past work has evaluated deceptive cues during cycling by manipulating cycling performance of accompanying avatars (Ansdell et al., 2018; Jones et al., 2016; Williams et al., 2015), or by manipulating perceived cycling time (Radel, Brisswalter & Perrey, 2017), here we explore deceptive cues about the virtual cycling environment itself. That is, deceptive cues were delivered by visually manipulating a virtual environment during stationary cycling (providing illusory hills) while holding work rate constant. We hypothesized that deceptive visual cues would induce error between expectations of effort and actual effort and impact feel good/feel bad responses as well as perceived exertion. We hypothesized that the type of illusory hill would elicit opposite effects on exercise experiences. For example, seeing an illusory ascending hill (*i.e.,* expecting an increase in resistance—but resistance does not change) would result in higher affective valence (feel better) and lower perceived exertion (feel like you are working less hard) relative to a flat terrain condition. Whereas, seeing an illusory descending hill (expecting a decrease in resistance—but resistance does not change) would result in lower affective valence (feel worse) and higher perceived exertion (feel like you are working harder) relative to a flat terrain condition. Finally, we hypothesized that interoceptive accuracy would interact with condition (uphill, downhill, flat) although given lack of past exercise-specific evidence, no directional hypotheses were made.

## Materials & Methods

Twenty participants (*n* = 10 female) were recruited *via* university/community flyers, social media (Facebook/Twitter), and word of mouth. *A priori* sample size was calculated based on previous research that found a large effect (*η*2  = 0.14) of VR exergaming on affective valence during cycling (Glen et al., 2017). Therefore, we conservatively powered to detect a small-moderate effect (Cohen’s *f* = 0.20), finding that 18 participants would provide 80% power given our within-subject design (three conditions, 16 measurement points (four time blocks × four hill types)), repeated measures correlation of 0.6, and *α* = 0.05, as calculated using GPower3.1 (Faul et al., 2007). Accounting for ∼10–15% drop-out (equipment failure, discontinuation), this resulted in 20 participants in total. While linear mixed models were used to analyse our data, the literature recommends using power analyses based on repeated measures ANOVAs to determine the sample size required for a linear mixed model analysis when simulations are not possible (as can occur with a novel experimental question) (Guo et al., 2013).

Healthy volunteers aged between 18–65 years with normal (or corrected-to-normal) vision were eligible, with exclusions if they had contraindications to cardiovascular exercise (*via* the Adult Pre-Exercise Screening System (Norton, 2012)), current lower limb/back pain, diagnosed cognitive/psychological condition(s), or were taking medications affecting cognition/physical performance. Ethical approval was received from the University of South Australia Human Research Ethics Committee (ID200790). All participants provided written, informed consent.

**Figure 1:**
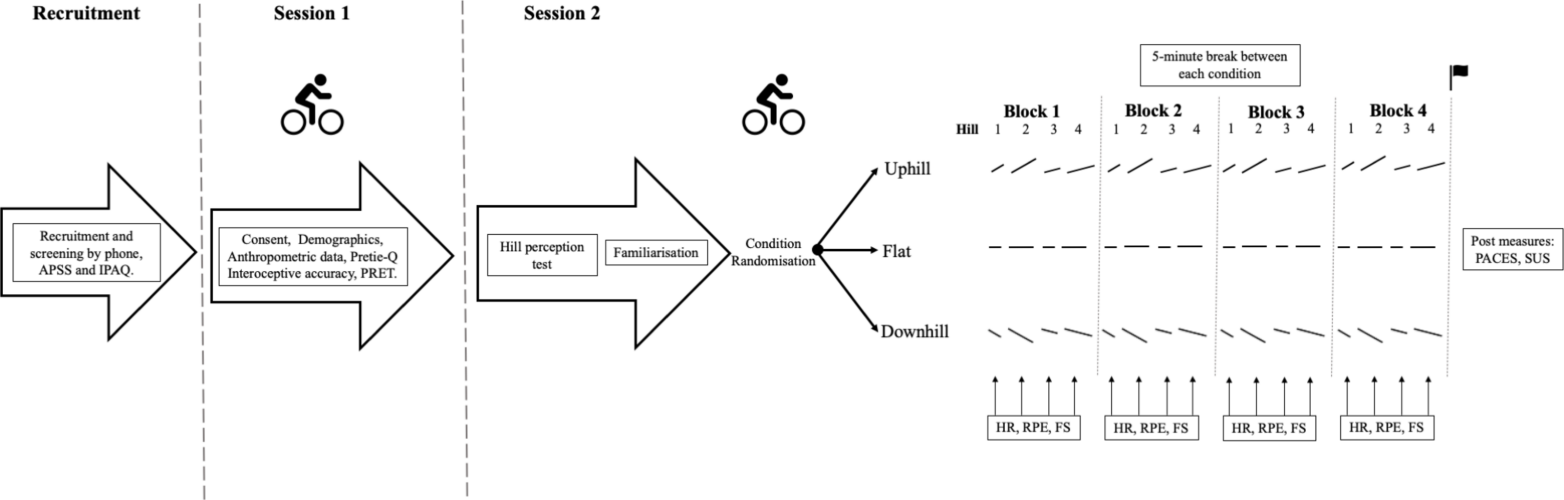
Study timeline and protocol overview. APSS, Adult Pre-Exercise Screening System; IPAQ, International Physical Activity Questionnaire; Pretie Q, The Preference for and Tolerance of the Intensity of Exercise Questionnaire; PRET, Perceptually Regulated Exercise Test; HR, Heart Rate; RPE, Rate of Perceived Exertion; FS, Feeling Scale; PACES, Physical Activity Enjoyment Scale; SUS, Slater-Usoh-Steed presence questionnaire.

Participants attended two sessions, one week apart (Fig. 1). In Session One, demographic (age, gender, height, weight) and exercise-relevant data, including Preference for and Tolerance of the Intensity of Exercise Questionnaire (Pretie-Q) (Ekkekakis, Hall & Petruzzello, 2005a) and current physical activity *via* International Physical Activity Questionnaire short form (IPAQ-SF) (Dinger, Behrens & Han, 2006) were collected. Interoceptive accuracy was evaluated at rest using an established heart rate detection task (Georgiou et al., 2015), where participants estimated their number of heart beats over time intervals. Heart rate was measured using a SphygmoCor system (Sydney, NSW, Australia) with single lead electrocardiogram. After sitting for 3 min, participants underwent a 10 second practice trial, followed by three formal trials (25, 35, and 45 seconds; order randomised, repeated twice), each separated by a 20 second break. Interoceptive accuracy was calculated as the mean absolute accuracy of the six measures (Georgiou et al., 2015), resulting in a continuous value between 0 and 1 where higher values represent greater interoceptive accuracy.

In Session One, participants also performed a 15-minute submaximal, perceptually-regulated exercise test (PRET) (Eston et al., 2008) involving an incrementally graded protocol using Borg’s 6-20 RPE scale, where 6 = ‘no exertion at all’ and 20 = ‘maximal exertion’ (Borg, 1982; Rejeski, 1985; Soriano-Maldonado et al., 2014). Five consecutive three-minute stages were undertaken at RPEs 9, 11, 13, 15, and 17 (Eston et al., 2008; Evans et al., 2015; Evans, Parfitt & Eston, 2013). The TrueOne 2400 (Parvo Medics, Salt Lake City, UT, USA) metabolic system measured pulmonary ventilation (VE) and gas exchange variables (oxygen uptake (VO2), carbon dioxide production (VCO2), ventilatory equivalent for oxygen (VE/VO2) and carbon dioxide (VCO2)), allowing estimation of ventilatory threshold (VT) using a triangulation of the modified v-slope method on PRET data (Gaskill et al., 2001). The cycling parameters (resistance, gearing, cadence) occurring at the time point corresponding to VT were used to standardise exercise intensity at Session Two. This intensity was chosen because between-person affective responses are most variable at VT, and not too high an intensity that interoceptive information would dominate affective responses, with little potential influence of exteroceptive information (Ekkekakis, Hall & Petruzzello, 2005b).

**Figure 2:**
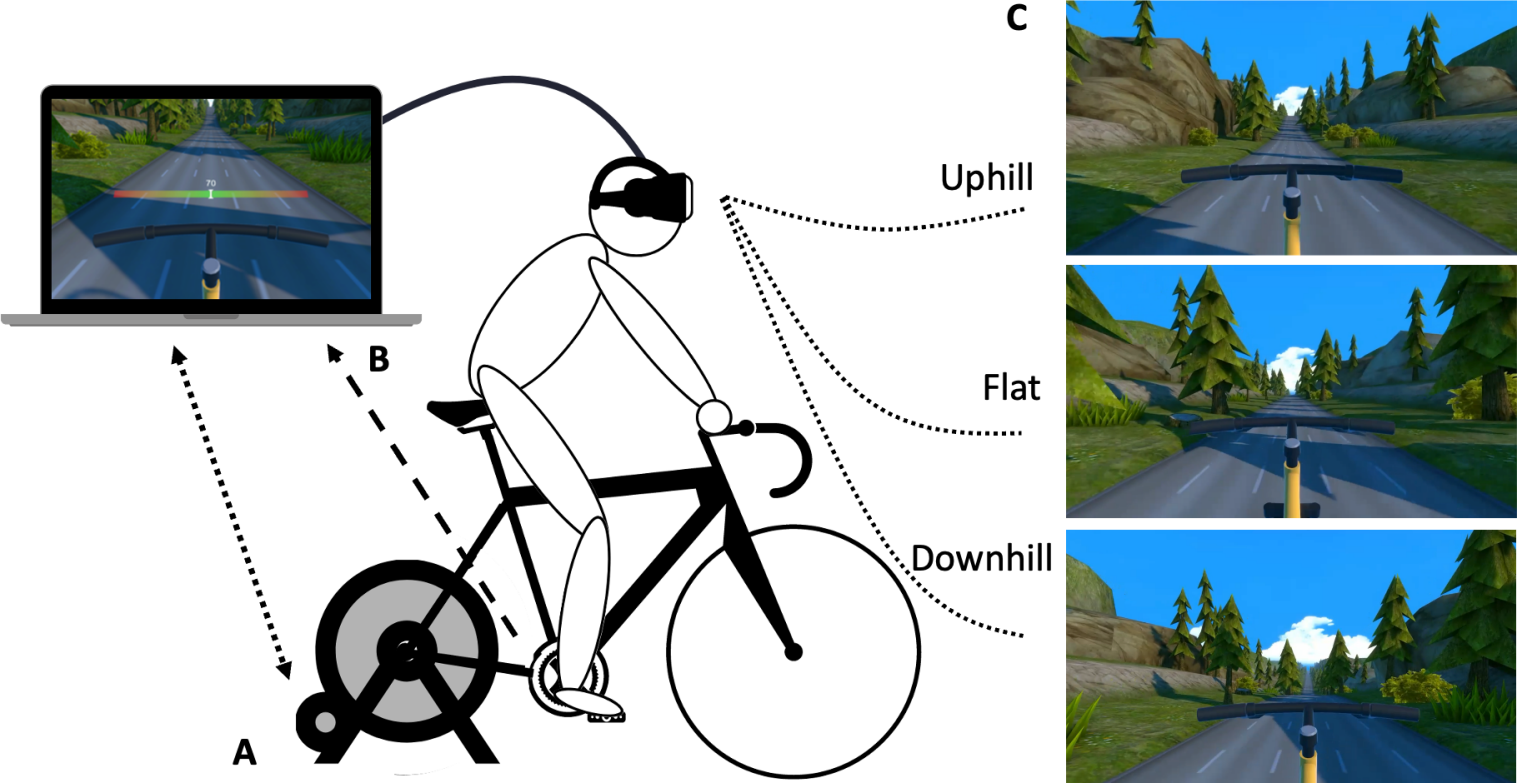
Customised virtual reality bike and experimental conditions. (A) Virtual reality bike set up, illustrating the stationary upright bike mounted to the Wahoo Kickr Smart Trainer, and the HTC Vive Pro VR head mounted display. The bidirectional dotted arrow represents communication *via* ANT+ wireless technology from the VR program to the Smart Trainer (controlling flywheel resistance) and from the Smart Trainer to the VR program (participant’s power output (Watts; per resistance and cadence) informing forward movement *via* track/scenery changes in the VR program). The unidirectional dotted arrow represents communication between the VR program and the Wahoo Cadence Sensor located on the right pedal. (B) Visual illustration of the cadence feedback indicator. While modelled on the computer, this indicator was also shown to participants in the head mounted display. Participants were instructed to keep their cadence in the green zone. The red zone indicates a pedal rate exceeding 5 revolutions/minute either side of pre-determined cadence. (C) Virtual reality experimental conditions of Illusory uphill, Flat terrain (control), and Illusory downhill. The cadence indicator has been removed from these visual depictions to facilitate illustration of the conditions but was always present during testing.

In Session Two, participants undertook a customised VR bike experience which involved cycling along a paved straight road in a forest. The VR set-up (Fig. 2A) used a stationary upright bike mounted to a Wahoo Kickr Smart Trainer (Wahoo Fitness LLC, Georgia, USA), an HTC Vive Pro VR head mounted display (HMD; New York, NY, USA), and a customised VR program *via* Steam VR (New York, NY, USA). The customised VR program allowed control of electromagnetic resistance applied to the trainer’s flywheel. Pedal cadence was measured *via* a cadence sensor (Wahoo Fitness LLC, Atlanta, GA, USA) attached to the right pedal, with real-time feedback provided *via* an indicator within the VR (Fig. 2B). Real-time, 360-degree tracking of head movement in the virtual environment was enabled *via* use of two infrared base stations that detected the Vive HMD sensor, and the VR bike set-up replicated reality *via* accurate height of the bike and position of rider on the bike.

All participants performed each of three VR cycling conditions—flat terrain, uphill terrain, downhill terrain—in a randomised, counterbalanced order (Fig. 2C). In the flat condition, participants viewed a flat road with changing peripheral scenery (*e.g.*, trees, grass) as they rode. In the illusory uphill condition, participants saw an identical road and changing peripheral scenery, but saw a randomised set of hill ascents, varying in length (150 m or 200 m) and gradient (10% or 15%), interspersed with flat sections of 100 m. The illusory downhill condition was identical, but used a randomised set of hill descents, interspersed with flat sections (see File S1 for video depicting the flat, uphill, and downhill terrain). Cadence was held constant across conditions through the real-time feedback provided by the indicator in the VR (Fig. 2B). The real-time feedback prompted users to increase or decrease pedal rate if it deviated by more than five revolutions/minute from their pre-determined cadence. Resistance was held constant within the VR program (*i.e.,* did not change with a virtual hill); participants were naïve to the lack of resistance change. Thus, exercise intensity was held constant within and between test conditions for each participant, with only vision-resistance coupling differing between conditions.

All conditions had four time blocks, each consisting of four hills of differing steepness and length (flat condition: same track length was used). The hills were provided in a pseudo-randomised, counterbalanced order between participants, with hill order held constant across each time block within a participant (Fig. 1). Affective valence was assessed *via* the Feeling Scale (−5 [very bad] to 0 [neutral] to +5 [very good]) (Van Landuyt et al., 2000) and perceived exertion *via* the Borg’s 6-20 RPE scale at the halfway point of each hill (or equivalent distance in the flat condition) using verbal response to scales visually presented within the HMD. Prior to completing the VR experimental conditions, a 3-minute warm-up cycle was undertaken to allow familiarisation with VR rating scale use and to confirm exercise intensity at VT (*via* HR/RPE at VT from Session 1). The three VR experimental conditions were then undertaken, each lasting a minimum of 10 min (total minimum distance: 3,300 m) and separated by a 5-minute break.

In addition to primary outcomes of affective valence and perceived exertion (described above), heart rate was measured using a Polar RS400 heart rate monitor (accuracy: ±1%; comparable to ECG; Engström et al., 2012) during each VR condition and was expressed as a percentage of heart rate measured at VT (estimated from the PRET using the Gaskill equation) (Gaskill et al., 2001). Heart rate was also monitored during rest to ensure sufficient recovery before undertaking the next VR condition. Power output (Watts) from the Wahoo Kickr (sampling rate: 4 Hz) was averaged for each time block. Following VR conditions, the Slater-Usoh-Steed presence questionnaire (SUS; higher scores reflect higher feelings of presence in the VR environment) (Riva, Davide & IJsselsteijn, 2003; Slater, Usoh & Steed, 1994) and the Physical Activity Enjoyment questionnaire (PACES; higher scores indicating greater enjoyment) (Jekauc et al., 2012; Kendzierski & De Carlo, 1991) were completed.

Linear mixed effects models regressed the outcomes of affective valence (FS; Model 1) and perceived exertion (RPE; Model 2) on the fixed effects of interoceptive accuracy, exteroceptive visual cues (Condition, 3 levels; Hill steepness, 2 levels; Hill distance, 2 levels), time (Block) and all two-way interactions with interoceptive accuracy. Random effect intercepts were fit for participants to account for repeated measures. A stepwise backwards selection process on fixed effects, using term significant at the *α* = 0.05 level, was used to simplify the initial model (all terms and two-way interactions with interoceptive accuracy) to the most parsimonious model while adhering to the principle of marginality (Nelder, 1977; Rawlings, Pantula & Dickey, 1998). Model fit within the stepwise procedure was performed using maximum likelihood; final models were refit using restricted maximum likelihood for unbiased parameter estimation (Bates et al., 2014; Boedeker, 2017). The Bayesian information criterion (BIC) statistic was produced in the backwards model selection to ensure improved goodness-of-fit with model reduction. Diagnostic plots of the final model were produced to ensure appropriate model fit. Analyses were performed in R, version 4.1.3 (R Core Team, 2022) with the packages lme4 (1.1-21) and lmerTest (3.1-2) (Bates et al., 2014; Kuznetsova, Brockhoff & Christensen, 2017). A Holm-Bonferroni correction was used to control for multiple comparisons (Holm, 1979). Effect sizes for fixed effects were provided *via* Cohen’s *f*^2^ which reflects the proportion of variance uniquely accounted for by the variable (and associated interactions for main effects), additional to all other model variables (Selya et al., 2012). To aid interpretation of interoceptive accuracy interactions, FS and RPE model predictions (with 95% confidence intervals) used lower and higher interoceptive accuracy values defined as one standard deviation below and above, respectively, the sample mean interoceptive accuracy.

## Results

Table 1 provides participant demographic and anthropometric data, with condition- and block-specific exercise outcomes in Table 2. There was <1% missing data for affective valence (7/960) and RPE (2/960) outcomes. Across all VR conditions, participants’ heart rate during the first minute of each condition was within 5% (mean: 3.6%; SD: 2.9%). In the final VR condition, participants’ heart rate was also within 5% of their baseline heart rate (mean: 3.8%; SD: 3.14%).

**Table 1:** Demographic, anthropometric and questionnaire data. Pretie Q, Preference for and tolerance of the Intensity of Exercise Questionnaire: preference for exercise intensity (higher scores indicate preference for lower intensity exercise). Tolerance for exercise intensity (higher scores indicate lower tolerance for intensity) IPAQ, International Physical Activity Questionnaire, assessing self-reported exercise, physical activity, and sedentary time per week; Slater-Usoh-Steed questionnaire for perceived presence of an experience (higher scores, greater sense of presence); bpm, beats per minute PACES, Physical activity enjoyment questionnaire (higher scores, greater enjoyment).

	*n*	**Min–Max**	**Mean ± SD**
Age (years)	20	20–61	30.2 ± 11.2
Height (cm)	20	160–184.5	170.6 ± 8.1
Weight (kg)	20	48.0–109.0	69.2 ± 18.8
BMI (kg/m^2^)	20	18.5–35.6	23.5 ± 4.8
Pretie-Q Intensity Preference	20	14–36	26.3 ± 5.5
Pretie-Q Intensity Tolerance	20	18–34	24.7 ± 4.3
IPAQ Vigorous intensity p/wk. (min)	20	0.0–630.0	113.3 ± 189.0
IPAQ Moderate intensity p/wk. (min)	20	0.0–1,680.0	345.0 ± 556.7
IPAQ Walking p/wk. (min)	20	30.0–2,520.0	338.5 ± 544.2
IPAQ Time sitting (hours)	19	3.0–15.0	7.3 ± 2.9
Slater-Usoh-Steed Questionnaire	19	11–34	24 ± 7.7
PACES	19	60–79	63.9 ± 17.8

**Table 2:** Performance and perceptual variables during virtual reality experimental conditions. Mean and SD for power (watts), affective valence (Feeling Scale; FS), ratings of perceived exertion (Borg’s RPE), heart rate (beats per minute; BPM), and total exercise time (minutes), organised by block and condition.

**Block**	**Downhill condition**	**Flat condition**	**Uphill condition**
**Power (Watts)**			
1	349.0 ± 128.3	354.4 ± 131.6	373.6 ± 142.5
2	338.5 ± 114.8	352.2 ± 120.6	358.8 ± 124.6
3	341.4 ± 114.6	348.7 ± 126.1	360.7 ± 125.8
4	341.0 ± 114.3	344.1 ± 122.0	358.7 ± 129.7
Total	1,369.9 ± 469.1	1,399.4 ± 498.2	1,451.9 ± 520.1
**Affective valence (FS)**			
1	3.1 ± 1.4	3.1 ± 1.5	3.2 ± 1.4
2	2.6 ± 1.8	2.9 ± 1.5	3.0 ± 1.4
3	2.3 ± 1.8	2.7 ± 1.8	2.8 ± 1.4
4	2.2 ± 1.9	2.4 ± 2.2	2.5 ± 1.6
Total	2.6 ± 1.7	2.8 ± 1.6	2.9 ± 1.3
**Perceived exertion (RPE)**			
1	10.6 ± 1.6	10.5 ± 1.8	10.3 ± 1.8
2	11.6 ± 1.6	11.3 ± 2.2	11.0 ± 1.9
3	12.2 ± 1.7	11.9 ± 2.5	11.5 ± 2.0
4	12.4 ± 2.2	12.1 ± 2.7	12.0 ± 2.4
Total	11.7 ± 1.6	11.4 ± 2.1	11.2 ± 1.9
**Heart rate (BPM)**			
1	128.4 ± 13.3	128.8 ± 13.3	129.1 ± 12.5
2	133.8 ± 15.7	135.6 ± 15.4	136.1 ± 13.4
3	137.3 ± 16.2	137.8 ± 15.5	138.6 ± 14.3
4	138.6 ± 16.9	139.4 ± 16.4	139.9 ± 15.0
Block	134.6 ± 15.4	135.4 ± 15.0	136.0 ± 13.4
**Time riding (Minutes)**			
Total	10.28 ± 0.3	10.31 ± 0.3	10.25 ± 0.3

**Figure 3:**
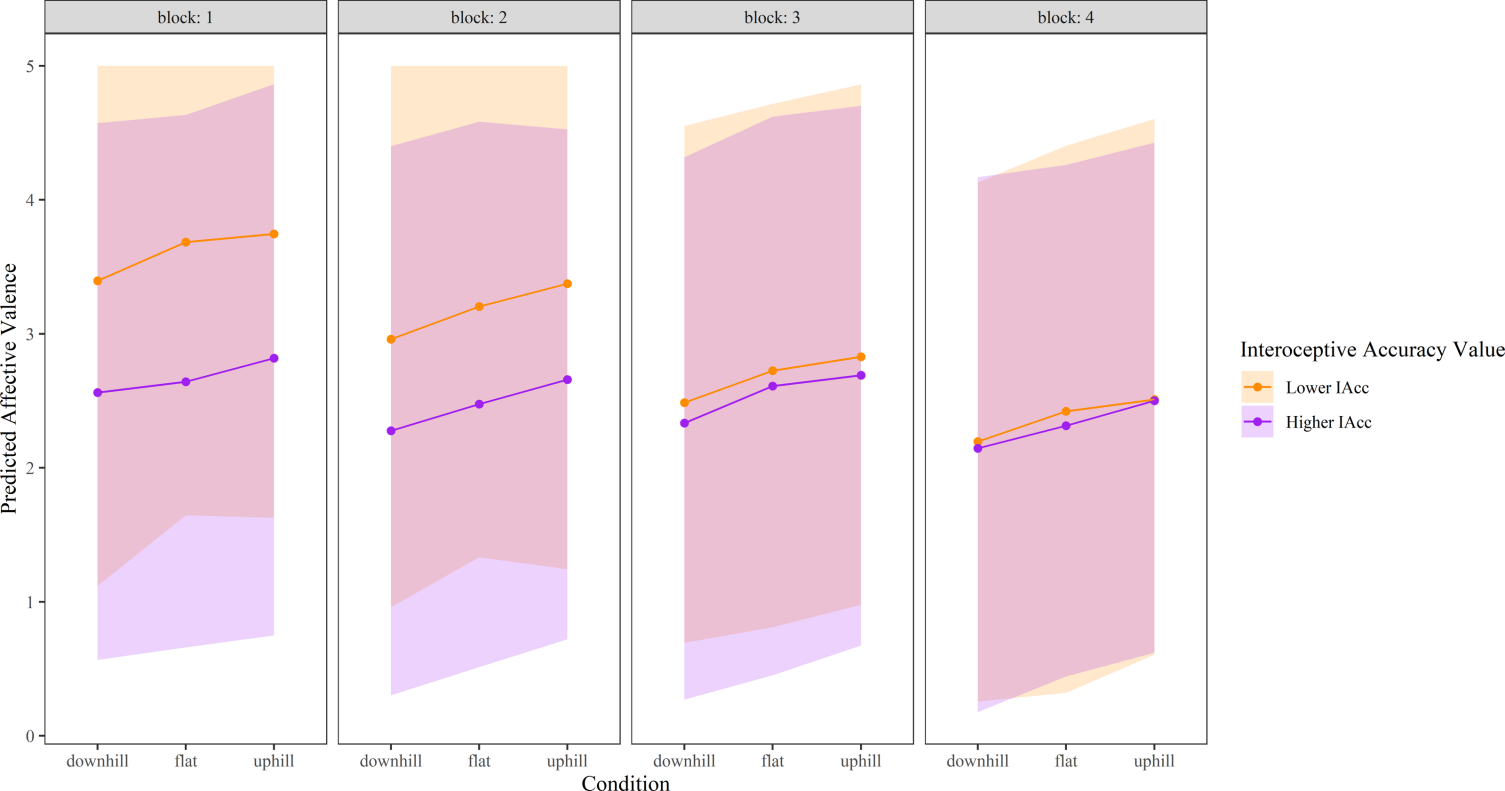
Affective valence over time in each experimental condition, modelled as a function of higher and lower interoceptive accuracy. ‘Higher’ interoceptive accuracy represents the mean interoceptive accuracy plus one SD, and ‘lower’ interoceptive accuracy was calculated as the mean interoceptive accuracy minus one SD. Shaded areas represent 95% confidence intervals. Affective valence was measured *via* the Feeling Scale (−5 to +5); given a lack of negative values within the data, only positive scale values are shown. IAcc, interoceptive accuracy.

For affective valence, there was a main fixed effect of Condition (*f*^2^ = 0.0198) showing lower affective valence during the downhill condition compared with the flat condition (Est = −0.21, t_925_ = −2.96, *p* = 0.003), but no difference between the uphill and the flat condition (Est = 0.11, t_925_ = 1.48, *p* = 0.14). There was no significant interaction between Condition and Interoceptive accuracy on affective valence, but there was a significant interaction between Interoceptive accuracy and Block (*p* = 0.00000084, *f*^2^ = 0.0302). At the sample mean of Interoceptive accuracy, differential effects in affective valence were observed for Blocks 3 (Est = 3.17, t_925_ = 4.09, *p* = 0.000048) and 4 (Est = 3.83, t_925_ = 4.91, *p* = 0.0000011), when compared with Block 1. Higher levels of interoceptive accuracy were associated a relative maintenance of affective valence (FS) by Blocks 3 and 4 compared to Block 1, whereas lower levels of interoceptive accuracy result in a relatively larger decline in affective valence by Block 3 and 4 (Fig. 3).

**Figure 4:**
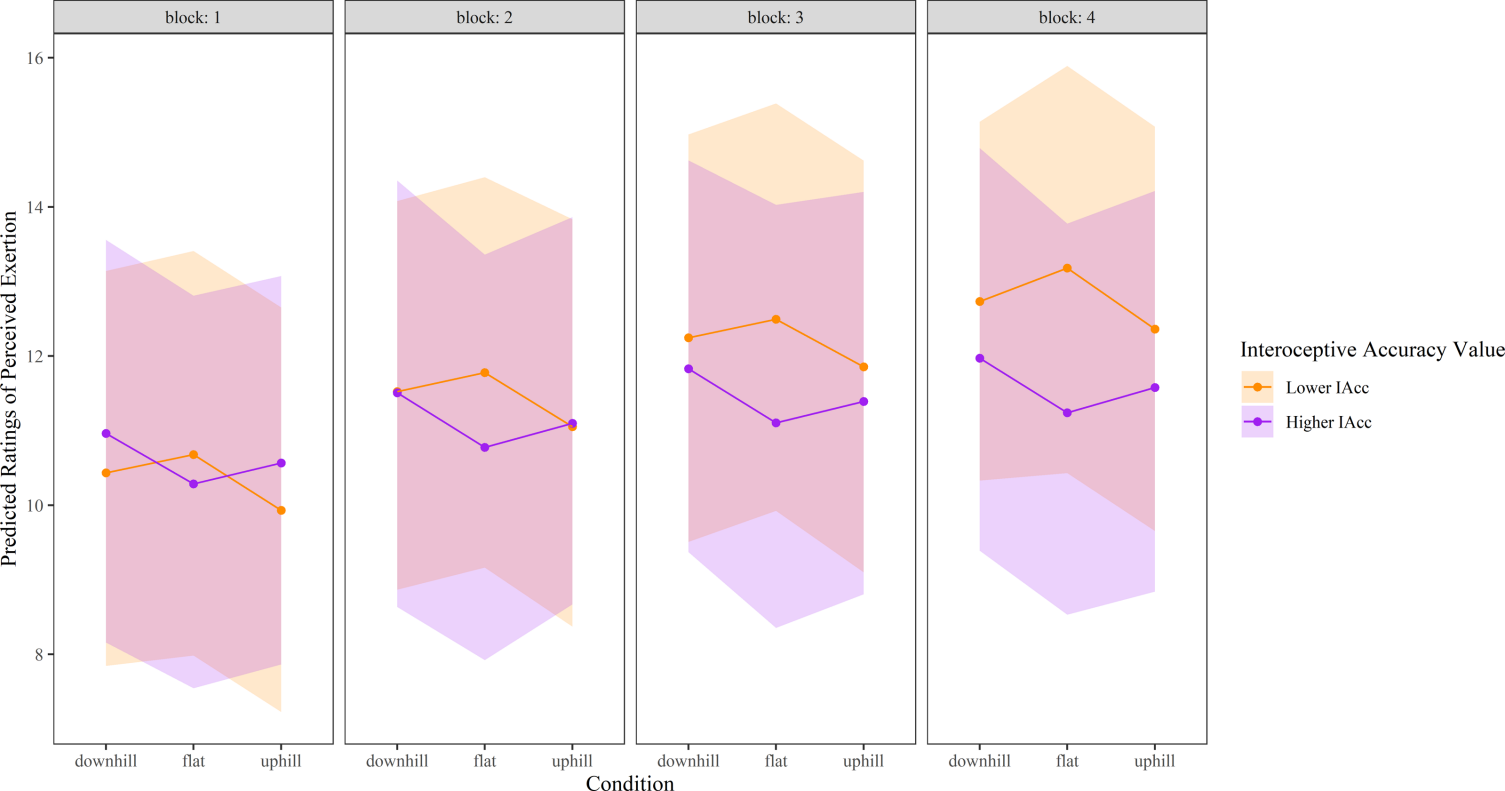
Perceived exertion ratings over time in each experimental condition, modelled as a function of higher and lower interoceptive accuracy. ‘Higher’ interoceptive accuracy represents the mean interoceptive accuracy plus one SD, and ‘lower’ interoceptive accuracy was calculated as the mean interoceptive accuracy minus one. Shaded areas represent 95% confidence intervals. Perceived exertion was measured using Borg’s Ratings of Perceived Exertion Scale (6 to 20). RPE, Ratings of perceived exertion.

For RPE, there was a significant interaction between both Condition (*p* = 0.00000024, *f*^2^ = 0.0307) and Block (*p* = 0.00000012, *f*^2^ = 0.0343) with Interoceptive accuracy. At the sample mean Interoceptive accuracy, RPE during both the downhill (Est = 4.25, t_922_ = 4.65, *p* = 0.0000037), and the uphill condition (Est = 4.51, t_922_ = 4.897, *p* = 0.0000012) differed from the flat condition. Higher levels of interoceptive accuracy were associated with a relative increase in RPE during both the uphill and downhill condition compared with the flat condition (Fig. 4). For the downhill condition, this appeared to drive an increase in RPE compared with flat in those with higher interoceptive accuracy, and a decrease in RPE compared with flat in those with lower interoceptive accuracy. In contrast, for the uphill condition, significant interactions appear driven by a large decrease in RPE compared with flat in those with lower levels of interoceptive accuracy, whereas those with higher levels of interoceptive accuracy had similar RPE values for uphill and flat. The significant interaction between Interoceptive accuracy and Block showed a consistent and growing reduction in RPE for increased sample scaled Interoceptive accuracy with Block 2 (Est = −2.98, t _922_ = −2.80, *p* = 0.005), Block 3 (Est = −4.53, t_922=_−4.28, *p* = 0.000021), and Block 4 (Est = −6.37, t_922_ = −5.98, *p* = 0.0000000032), compared to Block 1. Higher interoceptive accuracy levels resulted in relatively lower perceived exertion ratings in Blocks 2–4 when compared with Block 1, whereas lower levels of interoceptive accuracy resulted in relatively higher perceived exertion ratings in Blocks 2–4 (Fig. 4).

## Discussion

We aimed to determine the combined influence of deceptive visual cues about the environment and interoceptive accuracy on affective valence and perceived exertion during VR cycling, when exercise work rate was held constant. Our hypotheses that illusory ascending hills would be perceived as more pleasant and exertion perceived as lower than during a visually flat terrain (and vice versa for illusory descending hills) were partially supported, and in some cases, driven by interactions with interoceptive accuracy. In particular, contrary to our hypotheses, deceptive visual information (illusory uphill) did not improve affective valence. However, in support of our hypotheses, deceptive visual information negatively influenced affective valence (illusory downhill), and bi-directionally influenced perceived exertion, the latter specific to the visual context (type of illusory hill) and to participant-related factors (interoceptive accuracy). Together, these findings suggest that deceptive visual cues about the environment can influence exercise experiences, with the weighting given to these exteroceptive cues influenced by the accuracy with which interoceptive cues are detected.

Consistent with predictions from theories of perception and active inference (Friston, 2010; Seth & Friston, 2016), our results demonstrate that deceptive visual cues (illusory hills) can influence the perception of the exercise experience *itself* when an individual is denied opportunity to vary actual exercise effort. Past work has shown that deceptive exteroceptive visual cues can modulate exercise effort (*i.e.,* behavioural modification) while preserving perceptions of exertion (Williams et al., 2015). Our results suggest these constructs are bi-directionally interdependent: if one is not allowed (behaviour) the other is influenced (perception of how good/bad you feel).

Behavioral modification is often driven by principles of physiological homeostasis and detection of change in bodily state (Seth & Friston, 2016), with homeostatic threat most salient. Our work supports that homeostatic threat holds similar salience for perceptual shifts. During illusory descending hills, expected decreases in resistance did not occur, constituting a form of homeostatic threat based on violated expectations. These situations more consistently elicited perceptual shifts in exercise experiences than illusory ascending hills (easier than expected, thus minimal homeostatic threat). For example, relative to a flat surface, people experienced larger reductions in affective valence during illusory descending hills, but no increase in affective valence during illusory ascending hills. That is, it appears easier to make someone feel worse than better when using deceptive visual cues while cycling, particularly when they accurately detect internal bodily stimuli.

Recent theories suggest that affective valence is influenced by one’s ability to minimise ‘surprise’ and maintain physiological state within the bounds of homeostasis (Barrett & Simmons, 2015; Kiverstein, Miller & Rietveld, 2019). When individuals perceive a challenge to their homeostasis, such as visually seeing a hill to ascend, and it does not occur, they are likely able to minimise this threat to homeostasis quickly, leading to stable levels of affective valence. Conversely, if a person sees a descending hill and expects it to get easier, but it does not, the threat to homeostasis may be harder to minimise, resulting in ‘less positive’ (or negative) affective valence. Of interest, past work has explored a version of homeostatic threat in the context of deceptive cycling duration where participants were told they would cycle longer than in actuality (Radel, Brisswalter & Perrey, 2017). In contrast to our results, no effect of deceptive cycling duration on perceived exertion was found. These findings provide further support that the nature of homeostatic threat may hold importance to the effect on exercise experiences.

Previous work highlights the contribution of interoceptive accuracy to behavioral regulation during exercise (Herbert, Ulbrich & Schandry, 2007); our findings offer insight into its’ perceptual influences when exercise effort cannot vary. Here we show that interoceptive accuracy modulates the effect of deceptive visual cues on perceived exertion when exercising at VT. Consistent with the evolutionary value of detecting and responding to perceived threat (Friston, 2010), those with higher interoceptive accuracy show ‘protective’ but not ‘facilitatory’ perceptual shifts. Despite consistent physical effort, they perceive increased exertion in situations of homeostatic threat (illusory descending hills; effort is more than expected), but not reduced exertion in situations of homeostatic advantage (illusory ascending hills; effort is less than expected). Past work has shown that incongruence between sensory modalities can induce unpleasant feelings (Etzi et al., 2018; Furfaro et al., 2015). Thus, those with highly precise sensory detection may detect the incongruence between (visually-induced) *expected* effort and (interoceptively-confirmed) *actual* effort, blocking positive effects of homeostatic advantage (illusory ascending hills) and heightening negative impacts of homeostatic threat (illusory descending hills). In contrast, those with lower interoceptive accuracy exhibit only ‘facilitatory’ shifts. Regardless of the deceptive visual cue (ascending or descending hills), they perceive reduced exertion as compared to that experienced during flat terrain cycling. While such findings may suggest general attentional mechanisms underlie these effects, the flat control condition also involved immersive VR making a general mechanism of simple distraction unlikely. Furthermore, our finding of an absence of ‘protective’ shifts in perceived exertion for illusory downhills in those with lower interoceptive accuracy (does not feel harder), despite ‘protective’ shifts in affective valence (feels less ‘good’), cannot be easily explained and requires replication.

Past work demonstrates enhanced ability of exteroceptive cues to shift perceptual experiences when interoceptive accuracy is lower (Tsakiris, Jiménez & Costantini, 2011). Consistent with this, we found larger deceptive vision-induced shifts in perceived exertion (Fig. 4) and greater time-based decline in both affective valence and perceived exertion when interoceptive accuracy levels were lower. These results suggest a greater weighting of exteroceptive information, relative to interoceptive information, when the ability to accurately detect internal bodily signals is limited. During exercise, homeostatic threat is thought to be appraised by the increase in interoceptive cues (volume) that occurs when exercising, and by the comparison of the present state of physiological work with one’s capacity (Schandry, Bestler & Montoya, 1993). Such comparison requires precise information about physiological demand from interoceptive cues. Thus, one explanation for a greater reduction in affective valence and increase in RPE over time in those with lower levels of interoceptive accuracy may be due to less precision with which to detect interoceptive cues. Those with lower interoceptive accuracy may primarily rely upon the volume of incoming interoceptive (sensory) information, which increases over time, providing evidence that the threat to homeostasis is high. Those with higher interoceptive accuracy may be less influenced by the duration of exercise because they appraise physiological effort with more precision rather than relying on the volume of interoceptive cues alone.

Our findings may hold implications for exercise prescription and activity engagement. First, they support individualized exercise prescription. While people with lower levels of interoceptive accuracy are likely to respond positively to deceptive visual cues (particularly illusory ascending hills), avoiding use of deceptive visual cues in those with higher levels of interoceptive accuracy appears important. Second, improving interoceptive accuracy may be a relevant strategy to encourage maintenance of positive affect and more accurate perceptual responses to physiological demand with exercise. Visual heartbeat feedback training can improve interoceptive accuracy in individuals with (Schaefer et al., 2014) and without medical conditions (Meyerholz et al., 2019) although long-term impact and effects in the context of exercise are unknown. Finally, our findings suggest that VR may provide a means to alter automatic affective responses that occur with exercise (Brand & Ekkekakis, 2018). For example, increased affective valence during VR hill conditions in those with low interoceptive accuracy may represent a shift towards positive automatic affective valuation, promoting future exercise behaviours (Brand & Ekkekakis, 2018) Additionally, VR that uses incongruent stimuli (uphill but no increase in resistance) may help remodel an individual’s beliefs about anticipated affective experiences (*i.e.,* their past experience). While preliminary, these suggestions warrant further research.

Given the relatively small effects seen here, exploration of this VR paradigm using higher exercise intensities or in a clinical sample that experiences lower levels of affective valence during exercise may be useful. Our healthy, pain-free sample had relatively high levels of affective valence, which may result in a ceiling effect for enhancing exercise experiences. Further, exploring multisensory integration mechanisms may be fruitful to enhance exercise effects (Ernst & Bülthoff, 2004). For example, the addition of other sensory cues to the cycling experience—including naturalistic cues (enhancing the feeling of wind on the face providing information that you are moving faster) and non-naturalistic cues (rising pitch of sound while ascending hills)—may be relevant to explore. Such sensory manipulations may increase effects in those with lower interoceptive accuracy (Tsakiris, Jiménez & Costantini, 2011) or even help over-ride visual-interoceptive incongruence for those with higher interoceptive accuracy. While clearly speculative, further exploration holds merit.

The present study has several limitations. While powered for a small-moderate effect informed by previous literature (Glen et al., 2017), our sample size was relatively small, and our findings require replication. It is possible that null effects were driven by low power. Further, the heartbeat detection test (Schandry, 1981) has been critiqued given influence by confounding factors such as beliefs regarding heart rate, mental tracking, and counting strategies (Ring et al., 2015). Despite this, the measure has demonstrated links to behavioural and perceptual outcomes (Dunn et al., 2010; Tsakiris, Jiménez & Costantini, 2011) showing predictive value for behavioural responses related to bodily state (Herbert, Ulbrich & Schandry, 2007). Additionally, our VR program did not include a virtual body (*i.e.,* participants saw only bike handlebars and the surrounding environment, not a virtual avatar). Given embodiment of an avatar has been shown to have varying influence on subjective experience of virtual programs (Kim et al., 2020; Mouatt et al., 2020), this may be an important consideration in future VR development. Fourth, the age range for our sample was reasonably narrow (30.2 ± 11.2). Previous research has identified that cognitive resources may vary for different age groups and thus differentially impact engagement in physical activity (Cheval et al., 2020), therefore, it is unclear whether our results would generalise to younger or older populations. Future research investigating these effects across differing age groups is warranted.

While we based the duration of the between-condition rest period on pilot data and by monitoring our participant’s heart rate during rest, additional measures would have been useful. To establish that all participants recover to a standardised level, and to evaluate exercise effort more comprehensively during cycling, future research should consider using other physiological reactivity measures in addition to heart rate. These may include salivary cortisol, anxiety ratings, and/or measures of respiratory sinus arrhythmia and/or pre-injection period (index of parasympathetic and sympathetic influence, respectively, on the cardiac cycle) (Mücke et al., 2020; Salomon, 2020). Last, cycling at VT for three, 10-minute conditions in one session may have resulted in increasing fatigue over time, influencing affective valence and perceived exertion. Our use of randomised, counterbalanced VR conditions aimed to reduce condition-specific order effects due to fatigue and use of a standardised minimum exercise time aimed to avoid potential exercise duration-induced differences in fatigue. While power output was comparable across time (block) and condition, future work evaluating self-reported/neuromuscular fatigue, performing experimental conditions over numerous sessions, and using additional physiological reactivity measures to comprehensively capture exercise intensity is warranted. Finally, there is growing popularity of basic stationary cycling apps which have minimal coordination between environmental visual feedback and applied resistance (*i.e.,* effort and visual input may be incongruent). We did not formally screen for participants’ previous use of these apps, which is a limitation of our study, although that all participants completed all VR conditions means that any differences in exposure to these apps would not underlie between-condition effects. Future work should either recruit naïve participants or assess previous exposure to these apps to allow formal exploration of the effects of previous exposure on perceived effort/affective valence, or to control for previous exposure in analyses.

## Conclusions

Our findings showing that deceptive visual cues influence affective response and perception of effort during cycling, partially supported our hypotheses. Contrary to our hypotheses, deceptive visual cues did not improve affective valence. However, our findings provide preliminary evidence that visual information can be used to improve or worsen exercise experiences, depending on the visual context and on person-specific factors. Specifically, our findings demonstrate that the accuracy with which we perceive incoming body signals influences our exercise experiences and can induce differential effects on perceived exertion in response to deceptive visual information. Collectively, our study suggests that consideration of individual variance in interoceptive accuracy, and responses to exteroceptive cues, may provide insight into exercise strategies that result in lower perceptions of exercise effort. Further work is warranted to explore strategies to positively shift affective valence.

## Supplemental Information

10.7717/peerj.16095/supp-1 Supplemental Information 1 Virtual reality bike video Video showing all terrain types (flat, uphill, downhill)

10.7717/peerj.16095/supp-2 Supplemental Information 2 Analysis code

## Additional Information and Declarations

### Data Availability

The following information was supplied regarding data availability: The analysis code is available in the Supplementary File. The participants gave specific consent limited to the present project but did not consent to sharing the de-identified raw data publicly. The de-identified data are held in the UniSA Research Outputs Repository: Stanton, T, Mouatt, B, Smith, A, Parfitt, G, Smith, R, McDade, J, Stanford, T 2023, Exercise experiences during virtual reality cycling , University of South Australia, viewed 26 August 2023, retrieved from https://researchoutputs.unisa.edu.au/11541.1/a3b3ebd8-6778-4ba4-ab76-357ecd59e62f. The Preference for and Tolerance of the Intensity of Exercise Questionnaire (PRETIE-Q) is available at: https://faculty.sites.iastate.edu/ekkekaki/pretie-q.

## References

[ref-1] Andersen MM, Kiverstein J, Miller M, Roepstorff A (2022). Play in predictive minds: a cognitive theory of play. Psychological Review.

[ref-2] Ansdell P, Thomas K, Howatson G, Amann M, Goodall S (2018). Deception improves TT performance in well-trained cyclists without augmented fatigue. Medicine and Science in Sports and Exercise.

[ref-3] Barrett LF, Simmons WK (2015). Interoceptive predictions in the brain. Nature Reviews Neuroscience.

[ref-4] Bates D, Mächler M, Bolker B, Walker S (2014). Fitting linear mixed-effects models using lme4.

[ref-5] Boedeker P (2017). Hierarchical linear modeling with maximum likelihood, restricted maximum likelihood, and fully Bayesian estimation. Practical Assessment, Research, and Evaluation.

[ref-6] Borg GA (1982). Psychophysical bases of perceived exertion. Medicine & Science in Sports & Exercise.

[ref-7] Brand R, Ekkekakis P (2018). Affective–reflective theory of physical inactivity and exercise. German Journal of Exercise and Sport Research.

[ref-8] Chater N, Oaksford M, Hahn U, Heit E (2010). Bayesian models of cognition. Wiley Interdisciplinary Reviews: Cognitive Science.

[ref-9] Cheval B, Orsholits D, Sieber S, Courvoisier D, Cullati S, Boisgontier MP (2020). Relationship between decline in cognitive resources and physical activity. Health Psychology.

[ref-10] Craig AD (2003). Interoception: the sense of the physiological condition of the body. Current Opinion in Neurobiology.

[ref-11] Craig AD (2004). Human feelings: why are some more aware than others?. Trends in Cognitive Sciences.

[ref-12] Critchley HD, Harrison NA (2013). Visceral influences on brain and behavior. Neuron.

[ref-13] Dinger MK, Behrens TK, Han JL (2006). Validity and reliability of the international physical activity questionnaire in college students. American Journal of Health Education.

[ref-14] Dunn BD, Galton HC, Morgan R, Evans D, Oliver C, Meyer M, Cusack R, Lawrence AD, Dalgleish T (2010). Listening to your heart: how interoception shapes emotion experience and intuitive decision making. Psychological Science.

[ref-15] Ekkekakis P, Hall EE, Petruzzello SJ (2005a). Some like it vigorous: Measuring individual differences in the preference for and tolerance of exercise intensity. Journal of Sport and Exercise Psychology.

[ref-16] Ekkekakis P, Hall EE, Petruzzello SJ (2005b). Variation and homogeneity in affective responses to physical activity of varying intensities: an alternative perspective on dose–response based on evolutionary considerations. Journal of Sports Sciences.

[ref-17] Engström E, Ottosson E, Wohlfart B, Grundström N, Wisén A (2012). Comparison of heart rate measured by Polar RS400 and ECG, validity and repeatability. Advances in Physiotherapy.

[ref-18] Ernst MO, Bülthoff HH (2004). Merging the senses into a robust percept. Trends in Cognitive Sciences.

[ref-19] Eston R, Lambrick D, Sheppard K, Parfitt G (2008). Prediction of maximal oxygen uptake in sedentary males from a perceptually regulated, sub-maximal graded exercise test. Journal of Sports Sciences.

[ref-20] Etzi R, Ferrise F, Bordegoni M, Zampini M, Gallace A (2018). The effect of visual and auditory information on the perception of pleasantness and roughness of virtual surfaces. Multisensory Research.

[ref-21] Evans HJ, Ferrar KE, Smith AE, Parfitt G, Eston RG (2015). A systematic review of methods to predict maximal oxygen uptake from submaximal, open circuit spirometry in healthy adults. Journal of Science and Medicine in Sport.

[ref-22] Evans HJ, Parfitt G, Eston RG (2013). The perceptually regulated exercise test is sensitive to increases in maximal oxygen uptake. European Journal of Applied Physiology.

[ref-23] Faul F, Erdfelder E, Lang A-G, Buchner A (2007). G* Power 3: a flexible statistical power analysis program for the social, behavioral, and biomedical sciences. Behavior Research Methods.

[ref-24] Fox J, Bailenson JN (2009). Virtual self-modeling: the effects of vicarious reinforcement and identification on exercise behaviors. Media Psychology.

[ref-25] Friston K (2010). The free-energy principle: a unified brain theory?. Nature Reviews Neuroscience.

[ref-26] Furfaro E, Bevilacqua F, Berthouze N, Tajadura-Jimenez A (2015). Sonification of virtual and real surface tapping: evaluation of behavior changes, surface perception and emotional indices. IEEE MultiMedia.

[ref-27] Garfinkel SN, Critchley HD (2016). Threat and the body: how the heart supports fear processing. Trends in Cognitive Sciences.

[ref-28] Garfinkel SN, Seth AK, Barrett AB, Suzuki K, Critchley HD (2015). Knowing your own heart: distinguishing interoceptive accuracy from interoceptive awareness. Biological Psychology.

[ref-29] Gaskill SE, Ruby BC, Walker AJ, Sanchez OA, Serfass RC, Leon AS (2001). Validity and reliability of combining three methods to determine ventilatory threshold. Medicine & Science in Sports & Exercise.

[ref-30] Georgiou E, Matthias E, Kobel S, Kettner S, Dreyhaupt J, Steinacker JM, Pollatos O (2015). Interaction of physical activity and interoception in children. Frontiers in Psychology.

[ref-31] Glen K, Eston R, Loetscher T, Parfitt G (2017). Exergaming: feels good despite working harder. PLOS ONE.

[ref-32] Guo Y, Logan HL, Glueck DH, Muller KE (2013). Selecting a sample size for studies with repeated measures. BMC Medical Research Methodology.

[ref-33] Herbert BM, Ulbrich P, Schandry R (2007). Interoceptive sensitivity and physical effort: implications for the self-control of physical load in everyday life. Psychophysiology.

[ref-34] Higgins ET (2006). Value from hedonic experience and engagement. Psychological Review.

[ref-35] Holm S (1979). A simple sequentially rejective multiple test procedure. Scandinavian Journal of Statistics.

[ref-36] Jekauc D, Voelkle M, Wagner MO, Mewes N, Woll A (2012). Reliability, validity, and measurement invariance of the German version of the physical activity enjoyment scale. Journal of Pediatric Psychology.

[ref-37] Jones HS, Williams EL, Marchant DC, Sparks SA, Bridge CA, Midgley AW, Naughton LRMc (2016). Deception has no acute or residual effect on cycling time trial performance but negatively effects perceptual responses. Journal of Science and Medicine in Sport.

[ref-38] Jones L, Ekkekakis P (2019). Affect and prefrontal hemodynamics during exercise under immersive audiovisual stimulation: improving the experience of exercise for overweight adults. Journal of Sport and Health Science.

[ref-39] Kendzierski D, De Carlo KJ (1991). Physical activity enjoyment scale: two validation studies. Journal of Sport and Exercise Psychology.

[ref-40] Kim S-Y, Park H, Jung M, Kim K (2020). Impact of body size match to an avatar on the body ownership illusion and user’s subjective experience. Cyberpsychology, Behavior, and Social Networking.

[ref-41] Kiverstein J, Miller M, Rietveld E (2019). The feeling of grip: novelty, error dynamics, and the predictive brain. Synthese.

[ref-42] Knill DC, Pouget A (2004). The Bayesian brain: the role of uncertainty in neural coding and computation. Trends in Neurosciences.

[ref-43] Körding KP, Beierholm U, Ma WJ, Quartz S, Tenenbaum JB, Shams L (2007). Causal inference in multisensory perception. PLOS ONE.

[ref-44] Kuznetsova A, Brockhoff PB, Christensen RHB (2017). lmerTest package: tests in linear mixed effects models.

[ref-45] Meyerholz L, Irzinger J, Witthöft M, Gerlach AL, Pohl A (2019). Contingent biofeedback outperforms other methods to enhance the accuracy of cardiac interoception: a comparison of short interventions. Journal of Behavior Therapy and Experimental Psychiatry.

[ref-46] Morris JS (2002). How do you feel?. Trends in Cognitive Sciences.

[ref-47] Mouatt B, Smith AE, Mellow ML, Parfitt G, Smith RT, Stanton TR (2020). The use of virtual reality to influence motivation, affect, enjoyment, and engagement during exercise: a scoping review. Frontiers in Virtual Reality.

[ref-48] Mücke M, Ludyga S, Colledge F, Pühse U, Gerber M (2020). The influence of an acute exercise bout on adolescents’ stress reactivity, interference control, and brain oxygenation under stress. Frontiers in Psychology.

[ref-49] Nelder J (1977). A reformulation of linear models. Journal of the Royal Statistical Society: Series A.

[ref-50] Norton K (2012). New Australian standard for adult pre-exercise screening. Sport Health.

[ref-51] Radel R, Brisswalter J, Perrey S (2017). Saving mental effort to maintain physical effort: a shift of activity within the prefrontal cortex in anticipation of prolonged exercise. Cognitive, Affective, & Behavioral Neuroscience.

[ref-52] Rawlings JO, Pantula SG, Dickey DA (1998). Applied regression analysis: a research tool.

[ref-53] R Core Team (2022). https://www.R-project.org/.

[ref-54] Rejeski WJ (1985). Perceived exertion: an active or passive process?. Journal of Sport and Exercise Psychology.

[ref-55] Rhodes RE, Kates A (2015). Can the affective response to exercise predict future motives and physical activity behavior? A systematic review of published evidence. Annals of Behavioral Medicine.

[ref-56] Ring C, Brener J, Knapp K, Mailloux J (2015). Effects of heartbeat feedback on beliefs about heart rate and heartbeat counting: a cautionary tale about interoceptive awareness. Biological Psychology.

[ref-57] Riva G, Davide F, IJsselsteijn W (2003). Measuring presence: subjective, behavioral and physiological methods. Being there: concepts, effects and measurement of user presence in synthetic environments.

[ref-58] Salomon K (2020). Physiological reactivity. Encyclopedia of behavioral medicine.

[ref-59] Schaefer M, Egloff B, Gerlach AL, Witthöft M (2014). Improving heartbeat perception in patients with medically unexplained symptoms reduces symptom distress. Biological Psychology.

[ref-60] Schandry R (1981). Heart beat perception and emotional experience. Psychophysiology.

[ref-61] Schandry R, Bestler M, Montoya P (1993). On the relation between cardiodynamics and heartbeat perception. Psychophysiology.

[ref-62] Selya A, Rose J, Dierker L, Hedeker D, Mermelstein R (2012). A practical guide to calculating Cohen’s f2, a measure of local effect size, from PROC MIXED. Frontiers in Psychology.

[ref-63] Seth AK, Friston KJ (2016). Active interoceptive inference and the emotional brain. Philosophical Transactions of the Royal Society B: Biological Sciences.

[ref-64] Slater M, Usoh M, Steed A (1994). Depth of presence in virtual environments. Presence: Teleoperators & Virtual Environments.

[ref-65] Soriano-Maldonado A, Romero L, Femia P, Roero C, Ruiz J, Gutierrez A (2014). A learning protocol improves the validity of the Borg 6-20 RPE scale during indoor cycling. International Journal of Sports Medicine.

[ref-66] Tabor A, Thacker MA, Moseley GL, Körding KP (2017). Pain: a statistical account. PLOS Computational Biology.

[ref-67] Tabor A, Vollaard N, Keogh E, Christopher E (2019). Predicting the consequences of physical activity: an investigation into the relationship between anxiety sensitivity, interoceptive accuracy and action. PLOS ONE.

[ref-68] Tsakiris M, Jiménez AT, Costantini M (2011). Just a heartbeat away from one’s body: interoceptive sensitivity predicts malleability of body-representations. Proceedings of the Royal Society B: Biological Sciences.

[ref-69] Van Landuyt LM, Ekkekakis P, Hall EE, Petruzzello SJ (2000). Throwing the mountains into the lakes: on the perils of nomothetic conceptions of the exercise-affect relationship. Journal of Sport and Exercise Psychology.

[ref-70] Williams DM (2008a). Exercise, affect, and adherence: an integrated model and a case for self-paced exercise. Journal of Sport Exercise Psychology.

[ref-71] Williams DM, Dunsiger S, Ciccolo JT, Lewis BA, Albrecht AE, Marcus BH (2008b). Acute affective response to a moderate-intensity exercise stimulus predicts physical activity participation 6 and 12 months later. Psychology of Sport Exercise.

[ref-72] Williams EL, Massey H, Sparks S, Midgley AW, Marchant DC, Bridge CA, Mcnaughton LR (2015). Altered psychological responses to different magnitudes of deception during cycling. Medicine & Science in Sports & Exercise.

