# Supplementary material for: What I see and what I feel: the influence of deceptive visual cues and interoceptive accuracy on affective valence and sense of effort during virtual reality cycling
Source: PeerJ. 2023 Oct 4;11:e16095. doi: 10.7717/peerj.16095 (PMC10559878; doi:10.7717/peerj.16095)
Supplement: Supplemental Information 2 [file peerj-11-16095-s002.pdf]

Supplementary material

Analysis of affective valence (`affval`) and perceived exertion (`perexe`) outcomes

06 Feb 2023

Table of contents

Set up 2
- Packages 2
- Constants 3
- Functions 3

Data 6
- Import 6
- Wrangling 6
- Model predictions dataset 7

Modelling 8
- Outcome: `affval` 8
  - Stepwise selection and final model 8
  - Effect size calculations 11
  - Model predicted `affval` 13
- Outcome: `perexe` 17
  - Stepwise selection and final model 17
  - Effect size calculations 21
  - Model predicted `perexe` 22

R session information 25

Set up

Packages

```r
suppressPackageStartupMessages(suppressWarnings({

  library("readr")      # read data funcs
  library("dplyr")      # data manipulation
  library("tibble")     # improved data frames
  library("ggplot2")    # plotting
  library("purrr")      # apply functions over lists/vectors
  library("forcats")    # handling categorical variables
  library("tidyr")      # manipulate data to long/short representations

  library("mice")       # missing value utilities
  library("car")        # regression utilities

  library("lme4")       # linear mixed effects modelling
  library("merTools")   # alows prediction intervals using merMod objects
  library("lmerTest")   # step-wise lmer model selection

  library("knitr")      # pretty printing of tables: kable()
  library("gtsummary") # print summary tables of regression mods
  library("lattice")    # diagnostic plots

}))
```

Constants

```r
### plotting characters
# steep x long: "|"
# steep x short:  "/"
# less steep x long: "-"
# less steep x short: "\"
plchs <-
  c(
    "yes x long" = "|",
    "yes x short" = "/",
    "no x long" = "-",
    "no x short" = "\\"
  )

# plotting colour scheme
col_lohi <-
  c(
    "Lower IAcc" = "darkorange",
    "Higher IAcc" = "purple"
  )
```

Functions

The below functions make the calculation of Cohen's f^2 effect size statistic on `lme4::lmer()` (`merMod` class objects) possible. These functions are used later after models are fitted.

```r
# effect size (f^2), R^2 and residual variance functions

get_res_var_lmer <- function(lmer_obj) {
  return(sigma(lmer_obj) ^ 2)
}

get_lmer_r2 <- function(lmer_obj) {

  # residual variance of input model
  v_mod <- get_res_var_lmer(lmer_obj)

  # get formula for null model (intercept and REs only)
  null_form <- formula(lmer_obj, random.only = TRUE)
```

```r
  # create null model
  lmer_obj_null <- update(lmer_obj, null_form)

  # res var of null mod
  v_null <- get_res_var_lmer(lmer_obj_null)

  r2 <- (v_null - v_mod) / v_null

  attr(r2, "v_null") <- v_null
  attr(r2, "v_mod") <- v_mod

  return(r2)

}

rm_terms_lmer <- function(lmer_obj, terms) {

  update_form <- as.formula(paste0("~ . -", paste(terms, collapse = " - ")))
  print(update_form)

  lmer_obj_less_term <- update(lmer_obj, update_form)

  return(lmer_obj_less_term)
}


eff_size_f2 <- function(lmer_obj, terms) {

  r2_full <- get_lmer_r2(lmer_obj)[1]
  r2_less_term <- get_lmer_r2(rm_terms_lmer(lmer_obj, terms))[1]

  f2 <- (r2_full - r2_less_term) / (1 - r2_full)

  return(f2)

}
```

Convenience function for tidy printing of data.

```r
# function that replaces repeated values in a vector with empty strings
# as the verbose redundancy is too busy in some cases
```

```r
rm_rpts <- function(x) {
  x <- as.character(x)
  nx <- length(x)
  rm_ii <- rep(FALSE, nx)
  for (i in 2:nx) {
    if (x[i - 1] == x[i])
      rm_ii[i] <- TRUE
  }
  x[rm_ii] <- ""
  return(x)
}
```

Data

Import

```
dat_col_spec <-
  cols(
    partic = col_integer(),
    affval = col_integer(),
    perexe = col_integer(),
    int_sens = col_double(),
    block = col_integer(),
    cond = col_character(),
    steep = col_character(),
    dist = col_character()
  )

# read in dataset
hill_dat <- read_csv("dat/seefeel-hill-dat.csv", col_types = dat_col_spec)

# have a peak
hill_dat
```

Wrangling

```
# make factor variables and default levels
hill_dat <-
  hill_dat %>%
  mutate(
    cond = factor(cond),
    cond = relevel(cond, ref = "flat"),
    steep = factor(steep),
    steep = relevel(steep, ref = "no"),
    dist = factor(dist),
    dist = relevel(dist, ref = "short"),
    block = factor(block)
  )

# NAs only present in outcome vars
# md.pattern(hill_dat, rotate.names = TRUE)
```

```r
### centring the int_sens variable for easier interpretation of model intercept terms
# NOTE: want average of average participant values
isc <-
  hill_dat %>%
  group_by(partic) %>%
  summarise(avg_int_sens = mean(int_sens))

# This is the mean ISC over participants
mean_isc <- isc %>% pull(avg_int_sens) %>% mean(.)
sd_isc <- isc %>% pull(avg_int_sens) %>% sd(.)

# now modify the ISC values in the data
hill_dat <-
  hill_dat %>%
  mutate(int_sens = int_sens - mean_isc)
```

Model predictions dataset

This step creates a dataset for predictions from the models used later on.

```r
# create a minimal prediction dataset
pred_dat <-
  hill_dat %>%
  distinct(cond, block) %>%
  # NB: this is the mean ISC as we centred this data previously
  mutate(int_sens = 0, partic = 1L)

pred_dat_isc_plus_sd <-
  pred_dat %>%
  mutate(int_sens = int_sens + sd_isc)

pred_dat_isc_less_sd <-
  pred_dat %>%
  mutate(int_sens = int_sens - sd_isc)

pred_dat <-
  bind_rows(pred_dat_isc_less_sd, pred_dat, pred_dat_isc_plus_sd) %>%
  as.data.frame(.)
```

Modelling

Outcome: `affval`

Stepwise selection and final model

```
hill_dat_affmod <- subset(hill_dat, !is.na(affval))

# largest potential model
m1 <-
  lmer(
    affval ~
      int_sens * (cond + steep + dist + block) +
      (1 | partic),
    data = hill_dat_affmod,
    REML = FALSE
  )
# summary(m1)

# elimination of non-significant effects
# partly thanks to code found at::
# https://www.rdocumentation.org/packages/lmerTest/versions/2.0-36/topics/step
s1 <- step(m1) # consider optional arguments: test = c("none", "Rao", "LRT", "Chisq", "F")

# look at the model reduction
print(s1)
```

```
Backward reduced random-effect table:

             Eliminated npar  logLik    AIC    LRT Df Pr(>Chisq)
<none>                    18 -1307.5 2651.0
(1 | partic)          0   17 -1820.1 3674.2 1025.3  1  < 2.2e-16 ***
---
Signif. codes:  0 '***' 0.001 '**' 0.01 '*' 0.05 '.' 0.1 ' ' 1

Backward reduced fixed-effect table:
Degrees of freedom method: Satterthwaite

               Eliminated  Sum Sq Mean Sq NumDF  DenDF F value    Pr(>F)
int_sens:cond           1  0.4492  0.2246     2 933.00  0.2722    0.7618
int_sens:dist           2  0.6639  0.6639     1 933.01  0.8041    0.3701
```

```
dist                     3  0.4016  0.4016     1 933.00  0.4859    0.4859
int_sens:steep           4  0.7588  0.7588     1 933.00  0.9178    0.3383
steep                    5  0.2503  0.2503     1 933.00  0.3025    0.5825
cond                     0 17.0005  8.5002     2 933.00 10.2672 3.885e-05 ***
int_sens:block           0 25.9216  8.6405     3 933.01 10.4367 9.309e-07 ***
---
Signif. codes:  0 '***' 0.001 '**' 0.01 '*' 0.05 '.' 0.1 ' ' 1

Model found:
affval ~ int_sens + cond + block + (1 | partic) + int_sens:block
```

```r
# plot of post-hoc analysis of the final model
# plot(s1)


# use REML for final fit... see the following links
# https://stats.stackexchange.com/questions/41123/reml-vs-ml-stepaic
# https://stats.stackexchange.com/questions/116770/reml-or-ml-to-compare-two-mixed-effects
# https://stats.stackexchange.com/questions/414551/forward-selection-with-mixed-model-usin
m1_final <-
  lmer(
    affval ~ int_sens + cond + block + int_sens:block +
      (1 | partic),
    data = hill_dat_affmod,
    REML = TRUE
  )
summary(m1_final)
```

```
Linear mixed model fit by REML. t-tests use Satterthwaite's method [
lmerModLmerTest]
Formula: affval ~ int_sens + cond + block + int_sens:block + (1 | partic)
   Data: hill_dat_affmod

REML criterion at convergence: 2628.4

Scaled residuals:
    Min      1Q  Median      3Q     Max
-4.8486 -0.4947  0.0079  0.4971  4.3849

Random effects:
 Groups   Name        Variance Std.Dev.
```

```
 partic   (Intercept) 2.0548   1.4334  
 Residual             0.8351   0.9138  
Number of obs: 953, groups:  partic, 20

Fixed effects:
                 Estimate Std. Error        df t value Pr(>|t|)    
(Intercept)       9.18774    0.32866  19.56035  27.955  < 2e-16 ***
int_sens         -4.09769    3.01158  18.95131  -1.361 0.189589    
conddownhill     -0.21401    0.07242 924.99970  -2.955 0.003202 ** 
conduphill        0.10722    0.07260 925.00232   1.477 0.140037    
block2           -0.30735    0.08379 925.00216  -3.668 0.000258 ***
block3           -0.52714    0.08370 925.00199  -6.298 4.65e-10 ***
block4           -0.80794    0.08397 925.00476  -9.622  < 2e-16 ***
int_sens:block2   1.16392    0.77901 925.00736   1.494 0.135488    
int_sens:block3   3.17178    0.77612 925.00438   4.087 4.75e-05 ***
int_sens:block4   3.83155    0.78079 925.01026   4.907 1.09e-06 ***
---
Signif. codes:  0 '***' 0.001 '**' 0.01 '*' 0.05 '.' 0.1 ' ' 1

Correlation of Fixed Effects:
            (Intr) int_sn cnddwn cndphl block2 block3 block4 int_:2 int_:3
int_sens     0.001                                                        
conddownhll -0.110  0.000                                                 
conduphill  -0.110  0.000  0.500                                          
block2      -0.128 -0.002  0.000  0.002                                   
block3      -0.128 -0.002  0.000  0.000  0.503                            
block4      -0.128 -0.002 -0.002  0.002  0.501  0.502                     
int_sns:bl2 -0.002 -0.130  0.000 -0.004  0.006  0.010  0.010              
int_sns:bl3 -0.002 -0.131  0.000 -0.001  0.010  0.010  0.010  0.505       
int_sns:bl4 -0.003 -0.130  0.002  0.005  0.010  0.010  0.012  0.502  0.504
```

```r
# term significance -- Type III Wald chi-square tests
car::Anova(m1_final, type = "III")
```

```
Analysis of Deviance Table (Type III Wald chisquare tests)

Response: affval
                Chisq Df Pr(>Chisq)    
(Intercept)  781.4956  1  < 2.2e-16 ***
int_sens       1.8514  1     0.1736    
cond          20.3573  2  3.797e-05 ***
```

```
block            99.5601  3  < 2.2e-16 ***
int_sens:block   31.0369  3  8.350e-07 ***
---
Signif. codes:  0 '***' 0.001 '**' 0.01 '*' 0.05 '.' 0.1 ' ' 1
```

```
# prettier printing of regression model
m1_final %>%
  tbl_regression(
    estimate_fun = function(x) sprintf("%2.2f", x),
    pvalue_fun = function(x) sprintf("%1.8f", x)
  ) %>%
  add_global_p(keep = TRUE) %>%
  as_gt(.)
```

Characteristic	Beta	95% CI[1]	p-value
int_sens	-4.10	-10.40, 2.21	0.17362526
cond			0.00003797
flat	—	—	
downhill	-0.21	-0.36, -0.07	0.00320211
uphill	0.11	-0.04, 0.25	0.14003749
block			0.00000000
1	—	—	
2	-0.31	-0.47, -0.14	0.00025820
3	-0.53	-0.69, -0.36	0.00000000
4	-0.81	-0.97, -0.64	0.00000000
int_sens * block			0.00000083
int_sens * 2	1.16	-0.36, 2.69	0.13548840
int_sens * 3	3.17	1.65, 4.69	0.00004755
int_sens * 4	3.83	2.30, 5.36	0.00000109

[1]CI = Confidence Interval

Effect size calculations

```
### testing and extracting model elements for f^2 calc
# terms(formula(m1_final, fixed.only = TRUE))
# summary(m1_final)
# summary(rm_terms_lmer(m1_final, "cond"))
# summary(rm_terms_lmer(m1_final, c("int_sens", "int_sens:block")))
```

```r
### test functions
# get_lmer_r2(m1_final)
# get_lmer_r2(rm_terms_lmer(m1_final, "cond"))
### need to consider higher level terms with main effects
# get_lmer_r2(rm_terms_lmer(m1_final, c("int_sens", "int_sens:block")))

# cond eff size
eff_size_f2(m1_final, "cond")
```

```
~. - cond
<environment: 0x000000003006cf70>

[1] 0.01979127
```

```r
(get_lmer_r2(m1_final) -
    get_lmer_r2(rm_terms_lmer(m1_final, c("cond")))) /
  (1 - get_lmer_r2(m1_final)) # manual check
```

```
~. - cond
<environment: 0x0000000026b6c268>

[1] 0.01979127
attr(,"v_null")
[1] 0.9641614
attr(,"v_mod")
[1] 0.8350603
```

```r
# int_sens eff size
eff_size_f2(m1_final, c("int_sens", "int_sens:block"))
```

```
~. - int_sens - int_sens:block
<environment: 0x000000002f9d8770>

[1] 0.03016223
```

```r
(get_lmer_r2(m1_final) -
    get_lmer_r2(rm_terms_lmer(m1_final, c("int_sens", "int_sens:block")))) /
  (1 - get_lmer_r2(m1_final))  # manual check
```

```
~. - int_sens - int_sens:block
<environment: 0x000000002587e0a8>

[1] 0.03016223
attr(,"v_null")
[1] 0.9641614
attr(,"v_mod")
[1] 0.8350603
```

```r
# interaction only eff size
eff_size_f2(m1_final, "int_sens:block")
```

```
~. - int_sens:block
<environment: 0x000000002fc19e20>

[1] 0.03016245
```

```r
(get_lmer_r2(m1_final) -
    get_lmer_r2(rm_terms_lmer(m1_final, "int_sens:block"))) /
  (1 - get_lmer_r2(m1_final))  # manual check
```

```
~. - int_sens:block
<environment: 0x0000000025b5be78>

[1] 0.03016245
attr(,"v_null")
[1] 0.9641614
attr(,"v_mod")
[1] 0.8350603
```

Model predicted `affval`

```r
# ?predict.merMod
# ?predict

### usage
# predict(
#   object, newdata = NULL, newparams = NULL,
#   re.form = ~0, # or NULL for no REs
#   random.only = FALSE, terms = NULL,
#   type = c("link", "response"), allow.new.levels = FALSE,
#   na.action = na.pass, ...
# )


# test the above centring claim
# hist(hill_dat$int_sens_cont)

pred_est <-
  predict(
    m1_final,
    newdata = pred_dat,
    re.form = ~0
  )


# see:
# https://cran.r-project.org/web/packages/merTools/vignettes/Using_predictInterval.html
# ?merTools::predictInterval

pred_ci_est <-
  merTools::predictInterval(
    merMod = m1_final,
    newdata = pred_dat,
    which = c("full", "fixed", "random", "all")[4],
    level = 0.95,
    n.sims = 1000,
    stat = "median",
    type = "linear.prediction",
    include.resid.var = TRUE, # TRUE for including
    # fix.intercept.variance = TRUE
    seed = 1234567890
  ) %>%
```

```
  dplyr::filter(effect == "fixed") %>%
  arrange(obs)

pred_dat_aff <-
  bind_cols(pred_dat, tibble(fit_analytic = pred_est), pred_ci_est) %>%
  as_tibble()

# cat("#### Min and max difference between analytic fit and bootstrp median is:\n")
# with(pred_dat_aff, min(fit_analytic - fit))
# with(pred_dat_aff, max(fit_analytic - fit))

# pred_dat_aff %>%
#   dplyr::select(cond, block, int_sens, fit, lwr, upr) %>%
#   kable(., digits = 2)

pred_dat_aff <-
  pred_dat_aff %>%
  mutate(
    ISC_value =
      ifelse(
        int_sens < (0 - sd_isc/2),
        "Lower IAcc", # mean(IAcc) - sd(IAcc)
        ifelse(
          int_sens > (0 + sd_isc/2),
          "Higher IAcc", # mean(IAcc) + sd(IAcc),
          "Mean IAcc" # mean(IAcc)
        )
      ),
    ISC_value = factor(ISC_value),
    ISC_value = relevel(ISC_value, ref = "Lower IAcc"),
    cond =  relevel(cond, ref = "downhill")
  )


# change likert scale data recorded as [1, 12] to [-5, 5]
likert_adj <- -6

pred_dat_aff %>%
  dplyr::filter(ISC_value != "Mean IAcc") %>%
  mutate(
    trunc_up = if_else(upr > 11, 11L, as.integer(NA)),
```

```
    upr = if_else(!is.na(trunc_up), 11, upr),
    fit = fit + likert_adj,
    lwr = lwr + likert_adj,
    upr = upr + likert_adj
  ) %>%
  ggplot(data = ., aes(x= factor(cond), y = fit, col = ISC_value, group = ISC_value)) +
  geom_ribbon(aes(ymax = upr, ymin = lwr, fill = ISC_value), alpha = 0.2, colour = NA) +
  geom_point() +
  geom_line() +
  facet_wrap( ~ block, ncol = 4, labeller = label_both) +
  theme_bw() +
  theme(text = element_text(family = "serif"), panel.grid = element_blank()) +
  scale_color_manual(values = col_lohi) +
  scale_fill_manual(values = col_lohi) +
  labs(
    y = "Predicted Affective Valence",
    x = "Condition",
    col = "Interoceptive\nAccuracy Value",
    fill = "Interoceptive\nAccuracy Value"
  )
```

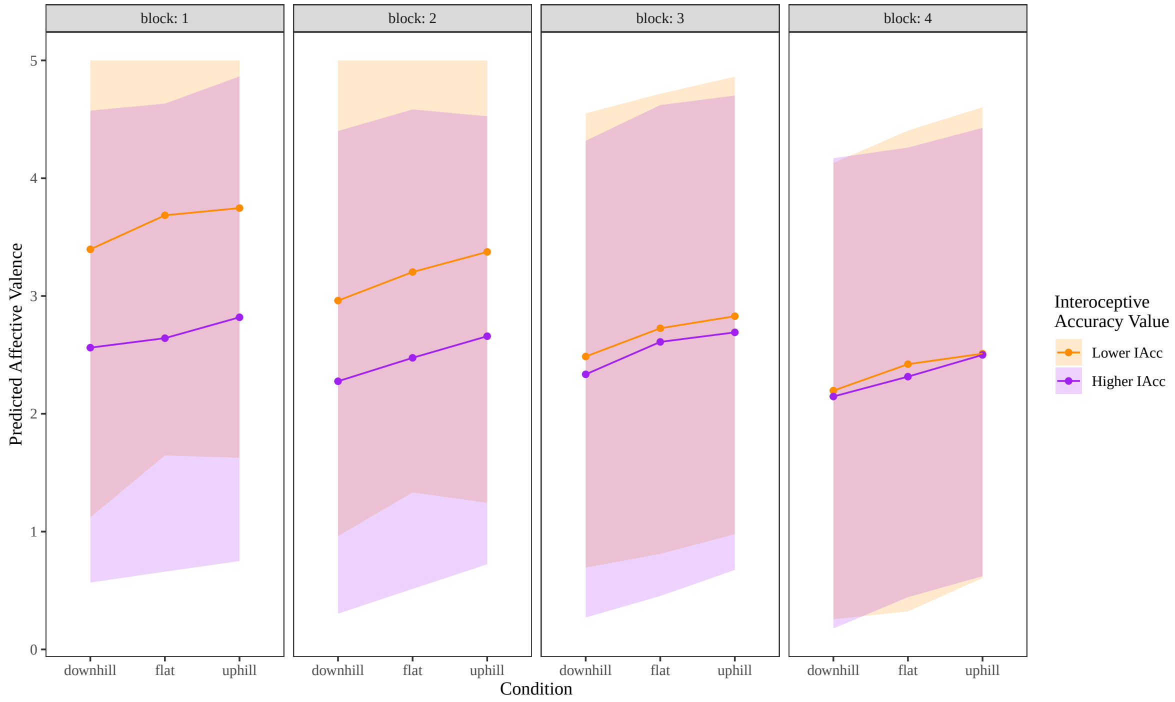

Outcome: perexe

Stepwise selection and final model

```
hill_dat_permod <- subset(hill_dat, !is.na(perexe))

# largest potential model
m2 <-
  lmer(
    perexe ~
      int_sens * (cond + steep + dist + block) +
      (1 | partic),
    data = hill_dat_permod,
    REML = FALSE
  )
# summary(m2)

# elimination of non-significant effects
s2 <- step(m2)

# look at the model reduction
print(s2)
```

```
Backward reduced random-effect table:

             Eliminated npar  logLik    AIC    LRT Df Pr(>Chisq)
<none>                     18 -1610.5 3257.0
(1 | partic)          0    17 -2048.8 4131.6 876.65  1  < 2.2e-16 ***
---
Signif. codes:  0 '***' 0.001 '**' 0.01 '*' 0.05 '.' 0.1 ' ' 1

Backward reduced fixed-effect table:
Degrees of freedom method: Satterthwaite

               Eliminated Sum Sq Mean Sq NumDF DenDF F value    Pr(>F)
int_sens:dist           1  0.058  0.0580     1   938  0.0376    0.8462
dist                    2  0.012  0.0125     1   938  0.0081    0.9282
int_sens:steep          3  0.265  0.2645     1   938  0.1718    0.6786
steep                   4  0.325  0.3250     1   938  0.2110    0.6461
int_sens:cond           0 47.519 23.7597     2   938 15.4232 2.568e-07 ***
int_sens:block          0 54.450 18.1501     3   938 11.7818 1.400e-07 ***
```

```
---
Signif. codes:  0 '***' 0.001 '**' 0.01 '*' 0.05 '.' 0.1 ' ' 1

Model found:
perexe ~ int_sens + cond + block + (1 | partic) + int_sens:cond + int_sens:block
```

```r
# use REML for final fit
m2_final <-
  lmer(
    perexe ~ int_sens + cond + block +
      int_sens:cond + int_sens:block +
      (1 | partic),
    data = hill_dat_permod,
    REML = TRUE
  )
summary(m2_final)
```

```
Linear mixed model fit by REML. t-tests use Satterthwaite's method [
lmerModLmerTest]
Formula: perexe ~ int_sens + cond + block + int_sens:cond + int_sens:block +
    (1 | partic)
   Data: hill_dat_permod

REML criterion at convergence: 3223.2

Scaled residuals:
    Min      1Q  Median      3Q     Max
-4.7274 -0.6103 -0.0234  0.6248  2.8202

Random effects:
 Groups   Name        Variance Std.Dev.
 partic   (Intercept) 2.989    1.729
 Residual             1.557    1.248
Number of obs: 958, groups:  partic, 20

Fixed effects:
                       Estimate Std. Error        df t value Pr(>|t|)
(Intercept)            10.44367    0.39904  19.98855  26.172  < 2e-16 ***
int_sens               -1.82776    3.68550  19.98716  -0.496   0.6254
conddownhill            0.25032    0.09873 927.99928   2.535   0.0114 *
conduphill             -0.19832    0.09873 927.99933  -2.009   0.0449 *
```

```
block2                    0.84316    0.11416 928.00025   7.386 3.37e-13 ***
block3                    1.38066    0.11416 928.00025  12.094  < 2e-16 ***
block4                    1.71399    0.11416 928.00025  15.014  < 2e-16 ***
int_sens:conddownhill     4.29035    0.91235 927.99966   4.703 2.96e-06 ***
int_sens:conduphill       4.42798    0.91115 927.99868   4.860 1.38e-06 ***
int_sens:block2          -2.80558    1.05396 927.99999  -2.662   0.0079 **
int_sens:block3          -4.27832    1.05396 927.99999  -4.059 5.34e-05 ***
int_sens:block4          -6.00929    1.05396 927.99999  -5.702 1.59e-08 ***
---
Signif. codes:  0 '***' 0.001 '**' 0.01 '*' 0.05 '.' 0.1 ' ' 1

Correlation of Fixed Effects:
             (Intr) int_sn cnddwn cndphl block2 block3 block4 int_sns:cndd
int_sens      0.000
conddownhll  -0.123  0.001
conduphill   -0.123  0.000  0.499
block2       -0.143 -0.001 -0.002 -0.002
block3       -0.143 -0.001 -0.002 -0.002  0.502
block4       -0.143 -0.001 -0.002 -0.002  0.502  0.502
int_sns:cndd  0.001 -0.123  0.002  0.000 -0.002 -0.002 -0.002
int_sns:cndp  0.000 -0.124  0.000  0.000  0.000  0.000  0.000  0.499
int_sns:bl2  -0.001 -0.143 -0.002  0.000  0.003  0.003  0.003 -0.003
int_sns:bl3  -0.001 -0.143 -0.002  0.000  0.003  0.003  0.003 -0.003
int_sns:bl4  -0.001 -0.143 -0.002  0.000  0.003  0.003  0.003 -0.003
             int_sns:cndp int_:2 int_:3
int_sens
conddownhll
conduphill
block2
block3
block4
int_sns:cndd
int_sns:cndp
int_sns:bl2   0.000
int_sns:bl3   0.000        0.502
int_sns:bl4   0.000        0.502  0.502
```

```r
# term significance
car::Anova(m2_final, type = "III")
```

```
Analysis of Deviance Table (Type III Wald chisquare tests)
```

```
Response: perexe
                  Chisq Df Pr(>Chisq)
(Intercept)    684.9663  1  < 2.2e-16 ***
int_sens         0.2459  1     0.6199
cond            20.7084  2  3.186e-05 ***
block          257.4023  3  < 2.2e-16 ***
int_sens:cond   30.5175  2  2.362e-07 ***
int_sens:block  34.9684  3  1.237e-07 ***
---
Signif. codes:  0 '***' 0.001 '**' 0.01 '*' 0.05 '.' 0.1 ' ' 1
```

```r
# prettier printing of regression model
m2_final %>%
  tbl_regression(
    estimate_fun = function(x) sprintf("%2.2f", x),
    pvalue_fun = function(x) sprintf("%1.8f", x)
  ) %>%
  add_global_p(keep = TRUE) %>%
  as_gt(.)
```

**Characteristic**	**Beta**	**95% CI**[1]	**p-value**
int_sens	-1.83	-9.52, 5.86	0.61994148
cond			0.00003186
flat	—	—	
downhill	0.25	0.06, 0.44	0.01139471
uphill	-0.20	-0.39, -0.00	0.04485907
block			0.00000000
1	—	—	
2	0.84	0.62, 1.07	0.00000000
3	1.38	1.16, 1.60	0.00000000
4	1.71	1.49, 1.94	0.00000000
int_sens * cond			0.00000024
int_sens * downhill	4.29	2.50, 6.08	0.00000296
int_sens * uphill	4.43	2.64, 6.22	0.00000138
int_sens * block			0.00000012
int_sens * 2	-2.81	-4.87, -0.74	0.00790347
int_sens * 3	-4.28	-6.35, -2.21	0.00005337
int_sens * 4	-6.01	-8.08, -3.94	0.00000002

[1]CI = Confidence Interval

Effect size calculations

```r
### testing and extracting model elements for f^2 calc
# terms(formula(m2_final, fixed.only = TRUE))
# summary(m2_final)
# summary(rm_terms_lmer(m2_final, c("int_sens", "int_sens:block")))


# interaction int_sens:block only eff size
eff_size_f2(m2_final, "int_sens:cond")
```

```
~. - int_sens:cond
<environment: 0x000000002fae1930>

[1] 0.03066341
```

```r
(get_lmer_r2(m2_final) -
    get_lmer_r2(rm_terms_lmer(m2_final, "int_sens:cond"))) /
  (1 - get_lmer_r2(m2_final))  # manual check
```

```
~. - int_sens:cond
<environment: 0x00000000195754b8>

[1] 0.03066341
attr(,"v_null")
[1] 2.112033
attr(,"v_mod")
[1] 1.557121
```

```r
# interaction int_sens:block only eff size
eff_size_f2(m2_final, "int_sens:block")
```

```
~. - int_sens:block
<environment: 0x000000002bcb0c30>

[1] 0.03433652
```

```r
(get_lmer_r2(m2_final) -
    get_lmer_r2(rm_terms_lmer(m2_final, "int_sens:block"))) /
  (1 - get_lmer_r2(m2_final))  # manual check
```

```
~. - int_sens:block
<environment: 0x000000002e6003b8>

[1] 0.03433652
attr(,"v_null")
[1] 2.112033
attr(,"v_mod")
[1] 1.557121
```

Model predicted `perexe`

```r
# test the above centring claim
# hist(hill_dat$int_sens_cont)

pred_est <-
  predict(
    m2_final,
    newdata = pred_dat,
    re.form = ~0
  )



pred_ci_est <-
  merTools::predictInterval(
    merMod = m2_final,
    newdata = pred_dat,
    which = c("full", "fixed", "random", "all")[4],
    level = 0.95,
    n.sims = 1000,
    stat = "median",
    type = "linear.prediction",
    include.resid.var = TRUE, # TRUE for including
    # fix.intercept.variance = TRUE
    seed = 1234567890
  ) %>%
```

```r
  dplyr::filter(effect == "fixed") %>%
  arrange(obs)

pred_dat_per <-
  bind_cols(pred_dat, tibble(fit_analytic = pred_est), pred_ci_est) %>%
  as_tibble()

# cat("#### Min and max difference between analytic fit and bootstrp median is:\n")
# with(pred_dat_per, min(fit_analytic - fit))
# with(pred_dat_per, max(fit_analytic - fit))

# pred_dat_per %>%
#   dplyr::select(cond, block, int_sens, fit, lwr, upr) %>%
#   kable(., digits = 2)

pred_dat_per <-
  pred_dat_per %>%
  mutate(
    ISC_value =
      ifelse(
        int_sens < (0 - sd_isc/2),
        "Lower IAcc", # mean(IAcc) - sd(IAcc)
        ifelse(
          int_sens > (0 + sd_isc/2),
          "Higher IAcc", # mean(IAcc) + sd(IAcc),
          "Mean IAcc" # mean(IAcc)
        )
      ),
    ISC_value = factor(ISC_value),
    ISC_value = relevel(ISC_value, ref = "Lower IAcc"),
    cond =  relevel(cond, ref = "downhill")
  )


pred_dat_per %>%
  dplyr::filter(ISC_value != "Mean IAcc") %>%
  ggplot(data = ., aes(x= factor(cond), y = fit, col = ISC_value, group = ISC_value)) +
  geom_ribbon(aes(ymax = upr, ymin = lwr, fill = ISC_value), alpha = 0.2, colour = NA) +
  geom_point() +
  geom_line() +
  facet_wrap( ~ block, ncol = 4, labeller = label_both) +
```

```r
theme_bw() +
theme(text = element_text(family = "serif"), panel.grid = element_blank()) +
scale_color_manual(values = col_lohi) +
scale_fill_manual(values = col_lohi) +
labs(
  y = "Predicted Ratings of Perceived Exertion",
  x = "Condition",
  col = "Interoceptive\nAccuracy Value",
  fill = "Interoceptive\nAccuracy Value"
)
```

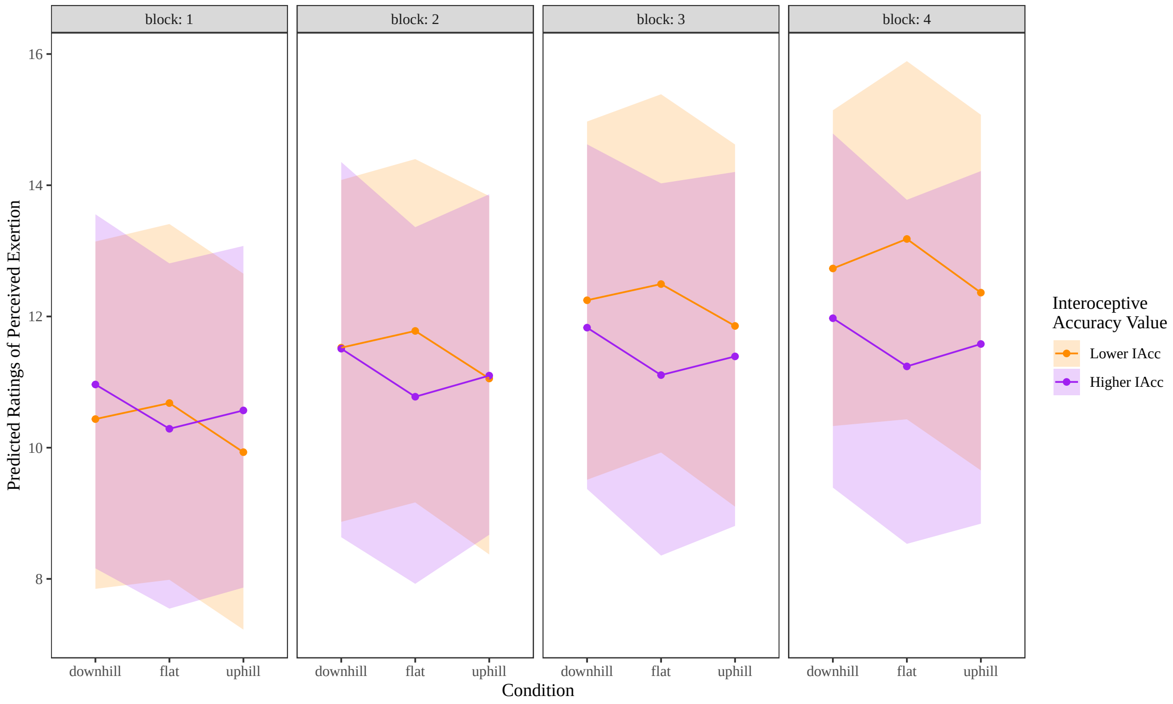

R session information

```r
# for reproducibility
sessionInfo()
```

```
R version 4.1.3 (2022-03-10)
Platform: x86_64-w64-mingw32/x64 (64-bit)
Running under: Windows 10 x64 (build 19044)

Matrix products: default

locale:
[1] LC_COLLATE=English_Australia.1252  LC_CTYPE=English_Australia.1252
[3] LC_MONETARY=English_Australia.1252 LC_NUMERIC=C
[5] LC_TIME=English_Australia.1252

attached base packages:
[1] stats     graphics  grDevices utils     datasets  methods   base

other attached packages:
 [1] lattice_0.20-45 gtsummary_1.6.1 knitr_1.37      lmerTest_3.1-3
 [5] merTools_0.5.2  arm_1.13-1      MASS_7.3-55     lme4_1.1-28
 [9] Matrix_1.5-3    car_3.0-12      carData_3.0-5   mice_3.15.0
[13] tidyr_1.2.0     forcats_0.5.1   purrr_0.3.4     ggplot2_3.4.0
[17] tibble_3.1.8    dplyr_1.0.10    readr_2.1.2

loaded via a namespace (and not attached):
 [1] bit64_4.0.5         vroom_1.5.7         jsonlite_1.8.0
 [4] splines_4.1.3       foreach_1.5.2       shiny_1.7.4
 [7] assertthat_0.2.1    broom.mixed_0.2.9.4 yaml_2.3.5
[10] globals_0.16.2      numDeriv_2016.8-1.1 pillar_1.8.1
[13] backports_1.4.1     glue_1.6.2          digest_0.6.29
[16] promises_1.2.0.1    minqa_1.2.4         colorspace_2.1-0
[19] htmltools_0.5.4     httpuv_1.6.8        pkgconfig_2.0.3
[22] labelled_2.9.1      broom_1.0.1         listenv_0.9.0
[25] haven_2.5.0         xtable_1.8-4        mvtnorm_1.1-3
[28] scales_1.2.1        later_1.3.0         tzdb_0.2.0
[31] farver_2.1.1        generics_0.1.3      ellipsis_0.3.2
[34] withr_2.5.0         furrr_0.3.0         cli_3.6.0
[37] crayon_1.5.2        magrittr_2.0.2      mime_0.12
[40] evaluate_0.20       future_1.30.0       fansi_1.0.4
```

```
[43] parallelly_1.34.0   broom.helpers_1.8.0 nlme_3.1-155
[46] tools_4.1.3         hms_1.1.1           lifecycle_1.0.3
[49] stringr_1.5.0       munsell_0.5.0       compiler_4.1.3
[52] rlang_1.0.6         blme_1.0-5          grid_4.1.3
[55] nloptr_2.0.3        gt_0.7.0            iterators_1.0.14
[58] rstudioapi_0.13     labeling_0.4.2      rmarkdown_2.20
[61] boot_1.3-28         gtable_0.3.1        codetools_0.2-18
[64] abind_1.4-5         DBI_1.1.2           R6_2.5.1
[67] bit_4.0.4           fastmap_1.1.0       utf8_1.2.2
[70] commonmark_1.8.1    stringi_1.7.12      parallel_4.1.3
[73] Rcpp_1.0.10         vctrs_0.5.2         tidyselect_1.2.0
[76] xfun_0.36           coda_0.19-4
```
